# Effect of Amino Acid, Sugar, Ca^2+^, and Mg^2+^ on Maillard Reaction-Associated Products in Modified Sparkling Base Wines During Accelerated Aging

**DOI:** 10.3390/molecules30030535

**Published:** 2025-01-24

**Authors:** Hannah M. Charnock, Gary J. Pickering, Belinda S. Kemp

**Affiliations:** 1 Department of Biological Sciences, Faculty of Mathematics & Science, Brock University, 1812 Sir Isaac Brock Way, St. Catharines, ON L2S 3A1, Canada; hannah.charnock@u-bordeaux.fr (H.M.C.); gpickering@brocku.ca (G.J.P.); 2 Cool Climate Oenology & Viticulture Institute, Brock University, 1812 Sir Isaac Brock Way, St. Catharines, ON L2S 3A1, Canada; 3 Gulbali Institute, Charles Sturt University, McKeown Drive, Wagga Wagga, NSW 2678, Australia; 4 Niab—National Institute of Agricultural Botany, New Rd, East Malling, Kent ME19 6BJ, UK

**Keywords:** sparkling wine, accelerated aging, Maillard reaction, metal ions, HS-SPME-GC/MS

## Abstract

The Maillard reaction (MR) between sugars and amino acids, peptides, or proteins is understood to occur gradually during the production and aging of sparkling wines, where it contributes to caramel, roasted, and toasted aromas. Divalent metal ions can accelerate the MR, although this has not been previously reported in wine or wine-like conditions. In this work, the effect of calcium (Ca) and magnesium (Mg) ions on the concentration of 10 Maillard reaction-associated products (MRPs) was measured in modified sparkling base wine during accelerated aging at 50 °C for four weeks. Chardonnay base wine was modified by the addition of fructose (0.02 M) and a single amino acid (lysine, glycine, cysteine; 0.01 M) in combination with Ca^2+^ or Mg^2+^ at zero, low (10 mg/L), or high (50 mg/L) dose levels. MRPs were quantified by headspace solid-phase microextraction coupled with gas chromatography–mass spectrometry (HS-SPME-GC/MS), sugar concentration was measured by enzymatic assay, and amino acids and free metal ions were monitored by capillary electrophoresis. Fructose levels did not substantially decrease during aging despite increases in all MRPs, suggesting that trace sugars or α-dicarbonyl species present in the wine matrix likely play a greater role in MRP formation than fructose. Aging duration and amino acid content had a greater effect than metal addition on the composition of the MRPs. Treatments containing cysteine and 50 mg/L Ca^2+^ had elevated concentrations of benzaldehyde and furfural ethyl ether following 4 weeks of accelerated aging. This work identified key MRPs that increase during base wine accelerated aging and informs future research on the relationship between wine composition and aging markers.

## 1. Introduction

The Maillard reaction (MR) has been widely studied in food science and is directly influenced by the physicochemical conditions of the system, including temperature, reaction time, pH, reactant composition (type and concentration), and metal ion content, among other factors [1,2]. The reaction is initiated by the non-enzymatic condensation of sugars with amino acids, peptides, or proteins, commencing a complex reaction cascade [1]. Maillard reaction-associated products (MRPs) including furan, thiazole, and pyrazine derivatives have been identified in aged traditional method (bottle-fermented/Méthode Champenoise) sparkling wines and are of key interest due to their desirable roasted, toasted, caramel, and nutty aroma qualities [3,4,5,6]. Despite the MR being favored at high temperatures [7], Maillard activity in sparkling wine occurs slowly during wine storage and likely proceeds only to intermediate stages due to the limiting low temperature (15 ± 3 °C), low pH (pH 3–3.4), and high acid (titratable acidity 7–12 g/L) conditions [8]. MRPs produced during the intermediate reaction stages are diverse, varying with the type and concentration of reactant species [8]. Strecker aldehydes and aromatic heterocycles containing nitrogen, sulfur, and oxygen develop at this stage, contributing to aromatic qualities [3,9]. Advanced stages of the Maillard reaction occur in high-heat conditions generally not relevant to sparkling wine and generate high molecular weight products including melanoidins and advanced glycation end products (AGEs) [10,11].

**Figure 1:**
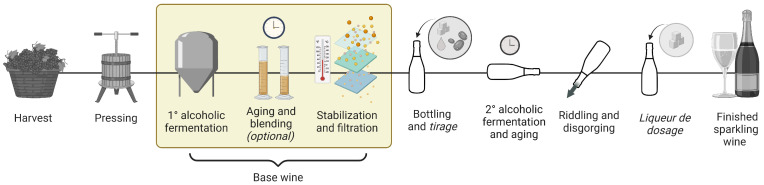
Sparkling wine production process with an emphasis on base wine stages.

During the production of traditional method sparkling wine, a primary alcoholic fermentation yields a still base wine that can undergo an optional aging period and/or blend with prior base wine vintages (i.e., reserve base wines) before bottling for the second alcoholic fermentation [12] (Figure 1). Various MRPs have been identified in sparkling base wines, including aromatic heterocycles and furan compounds [3,13]. The aging of reserve base wines is therefore a key production stage for the development of MRPs and is influenced by the composition of sugars and amino acids in the wine.

In base wine, fructose is the most abundant sugar (<1 g/L) due to the preferential metabolism of glucose by most *Saccharomyces cerevisiae* yeast strains [14]. In this study, Chardonnay base wine was modified by adding fructose in combination with a single amino acid (lysine, glycine, or cysteine) and divalent metal (Ca^2+^ or Mg^2+^) to investigate how these reaction precursors influence MRP formation during wine aging. This approach aimed to provide insights into the role of these precursors in shaping the chemical and volatile profiles of aged sparkling wines. Amino acids were selected based on their established role in the MR and inherent presence in base and sparkling wines [5]. Divalent metal ions (e.g., Mg^2+^, Ca^2+^, Fe^2+^, Cu^2+^, Al^2+^, Zn^2+^) were included due to their reported ability to accelerate the MR [15,16,17,18], although their specific influence on Maillard activity in wine has not yet been reported.

Metals in wine originate from both environmental and anthropogenic sources and include major and trace elements, with maximum permittable limits established by regional and international regulatory bodies [19,20]. At levels exceeding these limits, metals in wine can negatively impact sensory qualities, with increased browning, turbidity, astringency, and concerns for human health [19]. Potassium (K^+^), magnesium (Mg^2+^), calcium (Ca^2+^), and sodium (Na^+^) represent the major elements found in wine. Of these, Ca^2+^ and Mg^2+^ are the most abundant divalent metal ions and are found at similar concentrations, ranging from 50 to 150 mg/L [21,22,23,24,25,26]. However, factors such as grape variety, soil conditions, vineyard treatments, and winemaking practices can affect levels [26,27]. Consequently, Ca^2+^ and Mg^2+^ are central candidates for evaluating the influence of metal ions on the MR in wine.

Accelerated wine aging by heating has been applied in many finished wine and model wine studies to increase the rate of age-related reactions [28,29,30,31,32,33,34,35,36]. A summary of conditions utilized in the previous literature wine is presented in Table S1 (see Supplementary Materials) and informed the present study. Wines were heated to 50 °C for four weeks with sampling at 0-, 2-, and 4-week intervals. While increasing wine temperature is a convenient experimental approach compared to conventional aging, the composition of MRPs can diverge according to the reaction temperature [10,37,38,39]. Although this study was carried out in base wines subjected to accelerated aging, MRPs have also been shown to increase in finished sparkling wines during cellar aging conditions [4,6,9,40,41,42].

While model studies with controlled matrices have been widely used to investigate the influence of reactant composition, duration, and the presence of metal ions on the MR in wine and food systems [18,43,44,45], to our knowledge, this study represents the first use of a modified base wine matrix to examine MRP evolution in conditions that closely replicate those of sparkling wine. This novel approach enables a more accurate representation of the chemical matrix and interactions that can occur during aging. As such, this work offers a preliminary investigation into the MR in reserve base wines with a modified reactant composition and contributes new insights into how these changes influence MRP formation and the volatile profile of sparkling wines.

## 2. Results and Discussion

Base wine treatments were monitored during accelerated aging at 50 °C and included standard chemical parameters (pH, titratable acidity (TA), and browning), MR-active components (sugars, amino acids, and metals), and selected MRPs at intervals of 0, 2, and 4 weeks.

### 2.1. Standard Wine Chemical Parameters During Aging

**Figure 2:**
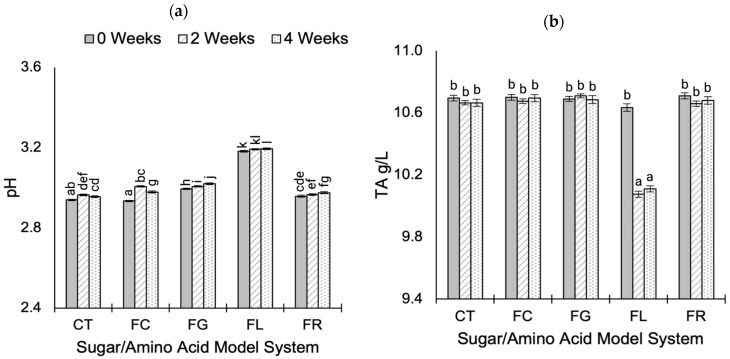
The pH (**a**) and TA (**b**) levels during the accelerated aging (at 50 °C) of modified Chardonnay base wine model systems for 4 weeks. Data represent the mean value ± standard deviation for all treatments prepared with the same sugar–amino acid combination (*n* = 10). Model system treatments include control (CT), fructose (FR), fructose-glycine (FG), fructose-lysine (FL), and fructose-cysteine (FC). Different letters represent significantly different means determined by Tukey’s post hoc test (*p* < 0.05).

Levels of pH and TA during aging are shown in Figure 2, grouped according to fructose–amino acid treatment (full data available in Supplementary Materials Tables S2 and S3). Treatment had the greatest effect on pH (*p* < 0.001; η^2^ = 0.943) and TA (*p* < 0.001; η^2^ = 0.571). The pH was slightly elevated in fructose-lysine wines (Figure 2a) and is attributed to the basicity of the lysine side chain. Additionally, in fructose-lysine wines, TA decreased by approximately 5% following 2 weeks of aging (Figure 2b). The reason for decreasing TA in fructose-lysine treatments is unclear and suggests organic acid utilization in side reactions or tartaric acid precipitation.

Minor pH differences with no discrete trends were identified during aging, and the slight variation in pH values (<0.08) suggests differences are unlikely to influence sensory perception, although sensory evaluation was not carried out in the present study. A previous study [38] of the MR in non-alcoholic model systems (heated at 100 °C for 240 min) reported a decrease in pH over time for reaction systems with an initial pH > 6, while pH remained relatively stable for conditions with an initial pH < 6. In addition, the authors highlighted the pH dependence for the reaction rate, with the MR proceeding more rapidly at a high pH [38]. This agrees with our findings, where low pH conditions (~pH 3) and the buffering capacity of the wine allow the pH to remain relatively stable during aging.

Accelerated MR activity under alkaline conditions has been widely reported and is primarily attributed to the enhanced reactivity of deprotonated amino acids, the proportion of which increases with pH [38,46,47]. Additionally, MR pathways proceed in a pH-dependent manner, with alkaline (pH > 7) conditions proceeding via a 2,3-enolization, while acidic conditions (pH < 7) undergo a 1,2-enolization [47]. These distinct pathways lead to distinct structural variations in α-dicarbonyl intermediates, impacting subsequent products in the reaction cascade [10,47].

The degree of browning index (A_420_) was measured to evaluate the effect of fructose–amino acid combinations and the presence of Ca^2+^ and Mg^2+^ ions at different dose rates (i.e., 10 mg/L or 50 mg/L) on yellow and brown pigment formation. A_420_ is a standard metric used to monitor MR activity and reaction kinetics in model systems [2,16], although oxidative browning in wine can interfere with this measurement. While the MR is likely to proceed only to intermediate stages in low temperature and low pH sparkling wine conditions [8], browning measurements were acquired due to the elevated temperature applied during accelerated aging, allowing the possible formation of pigmented intermediate MRPs. It is of note that not all intermediate MRPs necessarily contribute to browning during the reaction, as some may remain colorless. The interference of oxidative browning in A_420_ measurements was mitigated by heavily gassing the headspace of each reaction vessel with CO_2_ following sampling.

**Figure 3:**
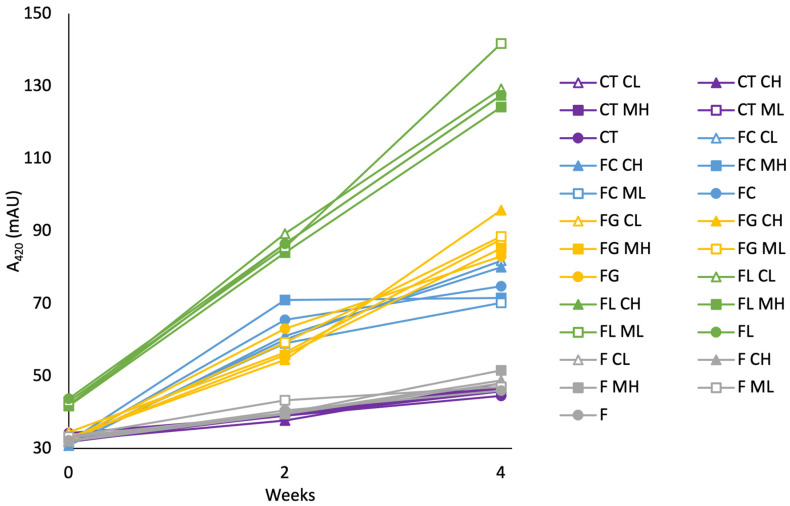
Development of browning (A_420_) in modified Chardonnay base wine model systems during 4 weeks of accelerated aging (50 °C), expressed as the mean value for each treatment (*n* = 2). Model system treatments include control (CT), fructose (F); fructose-glycine (FG), fructose-lysine (FL), and fructose-cysteine (FC), with metal additions of low (10 mg/L) Mg or Ca (ML or CL, respectively) and high (50 mg/L) Mg or Ca (MH or CH, respectively).

A_420_ levels increased relatively steadily for all treatments during aging (Figure 3). Reaction time and the fructose–amino acid treatment had the greatest effect on A_420_ levels (*p* < 0.001; η^2^ = 0.413 and 0.384, respectively). As expected, control (no fructose or amino acid) and fructose-only model systems were the least pigmented and had the slowest rate of browning. Comparatively, fructose-amino acid conditions facilitated both a higher rate and extent of pigment accumulation due to the presence of amino reaction precursors, enabling greater production of intermediate MRPs. Among the amino acids evaluated, lysine treatments exhibited the most pronounced browning, due to its ε-amino group which increases nucleophilicity and reactivity [44]. This is consistent with the previous literature, as lysine is a highly reactive amino acid in the MR, and is a key contributor to browning development in lysine-rich foods [18,44,48,49]. It is of note that immediately following base wine treatment preparations (0-week interval), fructose-lysine treatments showed elevated A_420_ due to the inherent yellow pigment of l-lysine. Despite this initial variance, the degree of browning increased at a greater rate for lysine treatments compared to both glycine and cysteine. Although moderate browning was observed for cysteine and glycine, the comparatively lower pigment development in cysteine treatments is consistent with earlier studies, suggesting that its sulfhydryl functional group acts to inhibit oxidative browning [50].

No systematic trends in browning were observed for treatments supplemented with calcium or magnesium, indicating that under the evaluated conditions, neither metal accelerated the rate of pigment accumulation during the MR. While the previous literature in simple MR model systems has shown that both Mg^2+^ and Ca^2+^ can drive MR pathways [15,16,17,18], the complex wine matrix along with different physicochemical factors including low pH, as well as differing reaction composition and ratios, may reduce their catalytic effect. Moreover, the interplay between various wine components (e.g., polyphenols, organic acids, sulfur compounds) may modulate or compete with MR pathways. Therefore, although these ions may be effective at driving MR activity in controlled conditions, their role in a winemaking context appears more nuanced.

### 2.2. Sugar Composition During Aging

**Figure 4:**
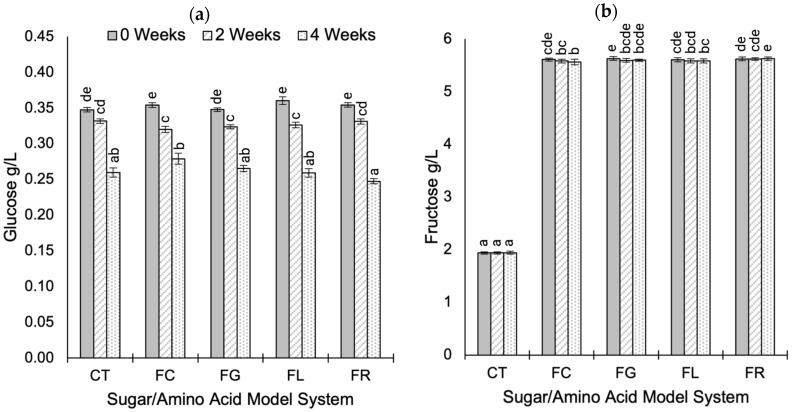
Fructose (**a**) and glucose (**b**) levels during the accelerated aging (at 50 °C) of modified Chardonnay base wine model systems for 4 weeks. Data represent the mean value ± standard error for all treatments prepared with the same sugar–amino acid combination (five treatments prepared in duplicate and measured in duplicate, *n* = 10). Model system treatments include control (CT), fructose (FR), fructose-glycine (FG), fructose-lysine (FL), and fructose-cysteine (FC). Different letters represent significantly different means determined by Tukey’s post hoc test (*p* < 0.05).

Glucose and fructose levels during accelerated aging are summarized in Figure 4, with full details in the Supplementary Materials (Tables S4 and S5). As anticipated, fructose–amino acid treatment was the primary source of variation in fructose levels (*p* < 0.001; η^2^ = 0.999), while the duration of accelerated aging had the greatest effect on glucose levels (*p* < 0.001; η^2^ = 0.772). No systematic trends were identified for metal additions or interaction effects.

Glucose levels were not adjusted in any treatments, and measured concentrations reflect residual amounts following the primary alcoholic fermentation. The glucose concentrations decreased in all wines during accelerated aging (Figure 4a), with a 0.10 g/L average decrease between 0 and 4 weeks.

Predictably, fructose concentrations differed according to the fructose–amino acid treatment, with control treatments containing the least residual fructose (0.35 ± 0.01 g/L) (Figure 4b). On average, fructose levels decreased by approximately 0.02 g/L between 0- and 2-week intervals, with no difference between week 2 and week 4. The absence of substantial fructose loss during accelerated aging has been previously reported in low pH conditions (heating at 100 °C for 120 min) since increasing pH leads to greater fructose utilization in MR pathways [46]. However, fructose is a precursor to the formation of many furan-derived MRPs in wine, whether via MR pathways containing fructose or via sugar degradation in acidic conditions [10,51], several of which were identified in these wines (Section 2.5). Notably, furan compounds may also be formed via interactions with trace sugars or α-dicarbonyl compounds present in base wine [2,45,52], and further research is required to understand the role of trace sugars on wine flavor development during aging.

Based on the limited consumption of glucose and fructose, other trace sugars found in sparkling base wine, including mannose, arabinose, fucose, galactose, and rhamnose [53,54], may have greater reactivity under acidic, low-temperature conditions, although they were not measured in the present study. For example, pentose sugars (e.g., ribose, xylose, arabinose) have a greater reactivity compared to hexose sugars (e.g., fructose, glucose) in model MR model systems [1,2]. This suggests that in principle, trace sugars could be involved in the formation of MRPs in wine, although their overall impact may be moderated by their low concentrations as well as interactions with other wine components. For example, the presence and abundance of many α-dicarbonyl compounds in wine (e.g., diacetyl, glyoxal, methylglyoxal, pentan-2,3-dione) suggest that Maillard interactions during wine aging may be, at least in part, the result of condensation reactions between α-dicarbonyl moieties and amino groups to initiate the reaction cascade [45,52]. α-Dicarbonyl compounds are more electrophilic than the carbonyl functional group of sugars in aqueous conditions at ambient temperature, supporting the likelihood of their involvement in the MR [45]. The role of α-dicarbonyls in Maillard pathways has been previously investigated in model sparkling wines (hydroalcoholic solutions of pH 3.5 and 8 at 25 °C), and included diacetyl, glyoxal, methylglyoxal, pentan-2,3-dione, acetoin, and acetol in combination with the amino acid cysteine [45]. These α-dicarbonyls are found at levels approximately ranging from sub-milligram to 5 mg/L in white wines [55,56], demonstrating their availability and reactivity, particularly in combination with the amino acid cysteine, although interactions were pH-dependent according to the α-dicarbonyl species [45]. Further research into Maillard interactions with trace residual sugars and α-dicarbonyl species is necessary to clarify their contribution to the MR during wine aging.

### 2.3. Amino Acid Composition During Aging

**Figure 5:**
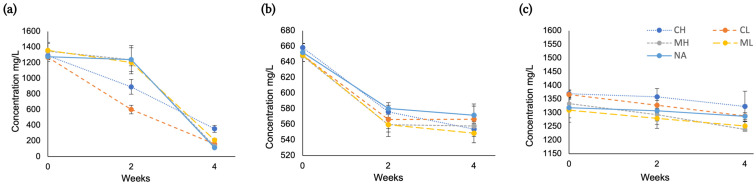
Concentration of free amino acids: (**a**) cysteine, (**b**) glycine, and (**c**) lysine measured in the respective modified Chardonnay base wine model systems containing fructose-cysteine, fructose-glycine, and fructose-lysine, respectively. Treatments included varying metal additions during accelerated aging (at 50 °C) for 4 weeks, with no metal addition (NA), low (10 mg/L) Mg or Ca (ML or CL), and high (50 mg/L) Mg or Ca (MH or CH). Each value is the mean of two model systems, analyzed in duplicate (*n* = 2). Error bars represent the standard deviation of the mean.

Free amino acids glycine, lysine, and cysteine were quantified by capillary electrophoresis (CE) in their respective fructose–amino acid treatments during aging, as shown in Figure 5, with full statistical analysis presented in Supplementary Materials (Table S6).

Lysine is one of the most abundant amino acids in traditional method sparkling wine, with reported concentrations between 32.6 and 133.6 mg/L, while glycine is found at lower levels ranging from 4.5 to 15.0 mg/L [57]. During the transformation of base wine to finished sparkling wine, the concentrations of lysine and glycine increase due to their release from autolyzed yeast cells throughout lees aging [58]. Conversely, cysteine is found at trace concentrations of 1.74–6.10 mg/L in reserve base wines [3], and levels decrease in finished sparkling wines to <0.1 mg/L, irrespective of lees aging time [58]. As such, all amino acids evaluated in this study have been reported in base wines during aging and are potentially available for MR interactions in non-modified conditions. However, concentrations utilized in these modified base wine conditions do not reflect amounts typically found in base wine but are exaggerated to identify trends in reactivity.

Cysteine and glycine were primarily influenced by aging duration (*p* < 0.001; η^2^ = 0.884 and 0.923, respectively), with concentrations decreasing over time. Treatments incorporating cysteine had the greatest overall change in amino acid concentration over 4 weeks, with an average 85% decrease (approximately 1114 mg/L; Figure 5a). Comparatively, glycine levels declined by an average of 14% over the same period (600 mg/L; Figure 5b). In lysine treatments, aging duration and the addition of Ca^2+^/Mg^2+^ had approximately equivalent effects on lysine levels (*p* < 0.001; η^2^ = 0.276 and 0.223, respectively), although no systematic trends were identified between calcium and magnesium, nor high or low (10 or 50 mg/L) metal levels. Lysine concentrations remained relatively stable for all fructose-lysine treatments (including Ca^2+^ and Mg^2+^ conditions), with a mean decrease of only 4% after 4 weeks of accelerated aging (53 mg/L; Figure 5c) despite these treatments having the greatest increase in browning. A possible explanation for this disparity is a limited reactivity of lysine towards Maillard intermediates under these physicochemical conditions. Lysine’s ε-amino group is likely to participate in early Maillard steps (e.g., Schiff base formation), potentially initiating the reaction cascade towards pigmented intermediates. However, if these intermediates do not continue to interact with lysine, this could lead to a limited depletion of overall lysine levels, despite more rapid browning development.

In fructose-cysteine treatments, a significant interaction between time × metal ion was identified (*p* < 0.001; η^2^ = 0.923). As shown in Figure 5a, the presence of Ca^2+^ was associated with a more rapid cysteine depletion in the first 2 weeks of aging, compared to Mg^2+^ and control conditions. Furthermore, low-dose Ca^2+^ (10 mg/L) treatments led to a more rapid decrease in cysteine compared to high-dose Ca^2+^ (50 mg/L) (Supplementary Table S7). We speculate that this could reflect stoichiometric effects, whereby a lower concentration of Ca^2+^ may form transient complexes with cystine more rapidly, facilitating reaction pathways, whereas higher Ca^2+^ levels could partially inhibit or divert these pathways.

Previous studies on the influence of metal ions in MR systems have demonstrated that Ca^2+^ and Mg^2+^ can accelerate the MR and browning (A_420_), with rates primarily influenced by the amino acid in the model solution [16,18]. It is of note that in these studies, the corresponding depletion of amino acids was not reported, and model conditions differ from the present study in terms of pH, matrix complexity, time, and temperature. Despite these differences, our results partially align with reported findings by demonstrating that elevated Ca^2+^ exerts a greater influence on cysteine than glycine or lysine. However, no systematic difference in A_420_ levels was observed for fructose-cysteine treatments containing Ca^2+^ additions, indicating that under highly acidic sparkling wine conditions, metal ions do not uniformly drive MR-related browning. Instead, the accelerated depletion of cysteine in the presence of Ca^2+^ appears to be more closely linked to the highly reactive structure of cysteine [18,45], which may preferentially interact with Ca^2+^ compared to Mg^2+^ under low pH and moderate temperature conditions. These findings highlight the complexity of extrapolating from model systems to real wine matrices and underscore the need for further research to clarify how divalent ions interact under actual sparkling wine conditions.

### 2.4. Ca^2+^ and Mg^2+^ During Aging

Free Ca^2+^ and Mg^2+^ concentrations were measured by CE accelerated aging, and ANOVA results are available in Supplementary Tables S8 and S9.

More common analytical techniques for the determination of metals in wine include ion chromatography, atomic absorption spectroscopy (AAS), atomic emission spectroscopy (AES), and inductively coupled plasma paired with mass spectroscopy or optical emission spectroscopy (ICP-MS or ICP-OES, respectively) [19,59]. CE has also been used to quantify metals in wine [60,61,62,63], offering rapid and simultaneous analysis of multiple elements in wine with low cost, low detection limits, and minimal sample preparation [64].

As anticipated, due to the high and low concentration treatments, metal additions were the primary contributor to variation in measured Ca^2+^ and Mg^2+^ concentrations (*p* < 0.001; η^2^ = 0.967 and 0.946 for Ca^2+^ and Mg^2+^, respectively). Aging time also contributed to variation in Ca^2+^ and Mg^2+^ levels (*p* < 0.001; η^2^ < 0.01), with a trend towards a slight decrease in measured free ions over time (Supplementary Table S9).

The interaction between time × metal × treatment was significant for Ca^2+^ levels (*p* < 0.001; η^2^ = 0.014) but not for Mg^2+^. Control wines treated with high levels (50 mg/L) of Ca^2+^ had an approximate 12.4% decrease (approximately 15 mg/L) in free Ca after 4 weeks, indicating that Ca^2+^ ions either precipitated or were partially bound during aging. Cations can form complexes with fructose in aqueous solutions [65]; however, since the control wines did not receive supplemental fructose, it is hypothesized that Ca^2+^ may preferentially bind with other trace sugars in the wine. Comparatively, Ca^2+^ levels did not decrease in control wines with low-dose Ca^2+^ (10 mg/L) nor in wines containing both fructose and Ca^2+^. Therefore, further research into the interactions between high levels of Ca and trace sugars is warranted.

In fructose-cysteine model systems, Ca^2+^ concentrations did not change during aging despite differences in the rate of cysteine depletion (Section 2.3). The reason for the more rapid depletion of cysteine in low Ca^2+^ treatments compared to high Ca^2+^ treatments remains unclear.

### 2.5. Maillard Reaction-Associated Products During Aging

Table 1 presents the results of ten volatile MRPs measured at intervals of 0, 2, and 4 weeks.

Various methods for quantifying heterocyclic MRPs have been proposed, most recently with an optimized headspace solid-phase microextraction coupled with gas chromatography–mass spectrometry (HS-SPME-GC/MS) [66]. This method was recently modified by our research group to analyze a targeted range of MRPs in traditional method sparkling wines [67] and has been applied in the present study to measure MRPs in modified base wines. Of the 22 MRPs included in the method, 10 were measured >LOQ.

#### 2.5.1. Influence of Time

**Figure 6:**
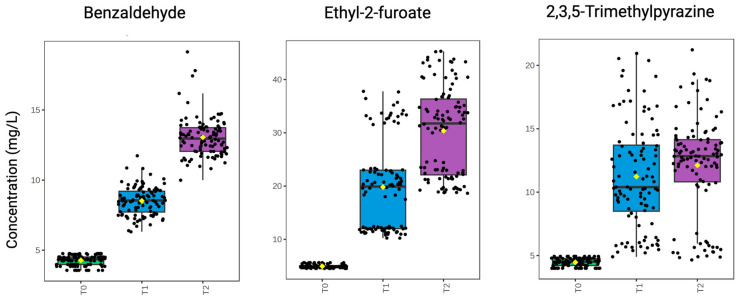
Box and whisker plots of Maillard reaction-associated products (mg/L) > LOQ at all time intervals (T0 = 0 weeks; T1 = 2 weeks; T2 = 4 weeks) during accelerated aging at 50 °C (*n* = 50). The upper and lower edges of boxes represent interquartile range from the 25th to 75th percentile, respectively; the internal horizontal line represents the median, and the yellow diamond indicates the mean. Whiskers above and below the boxes extend to the maximum and minimum values, respectively, with outliers signified as data points outside these bounds.

MRPs measured in the modified base wines unanimously increased over 4 weeks of accelerated aging. Benzaldehyde, ethyl-2-furoate, and 2,3,5-trimethylpyrazine were above their respective limits of quantification (LOQ) at all aging intervals and are visualized in Figure 6. Of the remaining MRPs, many were only measured >LOQ after 2 weeks of aging and included 2-acetylfuran, 5-methylfurfural, furfuryl ethyl ether, and 2,3-dihydrobenzofuran (Table 1). Thiazole levels remained <LOQ in control and fructose-only wines at 2 weeks but were quantified at week 4. Additionally, furfural was below its limit of detection (LOD) at the 0-week time interval in all wines. In fructose-cysteine wines, furfural levels remained <LOD at 2 weeks, and in fructose-cysteine wines with Ca^2+^ additions, furfural was <LOD at 4 weeks. Similarly, homofuraneol levels were <LOQ in all treatments at the initial 0-week timepoint and remained <LOQ in fructose-cysteine treatments at subsequent 2- and 4-week sampling points.

Table 2 presents results from a three-way ANOVA (time × treatment × metal ions). As previously mentioned, time was a significant factor (*p* < 0.001) in the concentration of all measured analytes, with levels increasing over the 4 weeks. Aging time was the greatest contributor to the evolution of benzaldehyde, 2,3,5-trimethylpyrazine, and ethyl-2-furoate (η^2^ > 0.5), all shown in Figure 6.

Benzaldehyde is derived from the oxidation of benzylic alcohol or during Maillard interactions with phenylalanine, an amino acid naturally present in sparkling base wines [45,58]. While phenylalanine was not added in the experimental conditions nor measured in the base wine, its inherent presence is likely, at least in part, to be responsible for the increase in benzaldehyde during aging. Benzaldehyde contributes a desirable bitter almond, sweet, buttery odor to wine [68,69] with an odor detection threshold (ODT) of 3–3.5 mg/L [70]. It was found above its ODT in all samples, with odor activity values (OAV) ranging from 3.6 in control wines up to 4.5 in fructose-cysteine treatments. The OAV, calculated as the ratio between the measured concentration of a compound and its reported ODT, infers the importance of an individual compound to the overall aroma profile. The cause of variation in benzaldehyde levels among fructose–amino acid treatments is unclear.

Comparatively, 2,3,5-trimethylpyrazine is produced in Maillard reactions involving glycine [71], and correspondingly, concentrations were elevated in fructose-glycine treatments (*p* < 0.05). This is discussed in detail in the section, “Fructose-Glycine Treatments”.

In addition, ethyl-2-furoate increased during accelerated aging, consistent with the previous literature on cellar-temperature (non-heated) aging of base and sparkling wines [13,72]. Ethyl-2-furoate is formed via furfural or furfuryl alcohol as intermediates in the MR [72]. However, in our experimental wines, furfural was <LOD until the 2-week interval for most treatments, suggesting that furfuryl alcohol and other furan compounds are likely greater contributors to its proliferation. To our knowledge, no sensory threshold information is available for ethyl-2-furoate.

#### 2.5.2. Influence of Treatment

The fructose–amino acid treatment and the interaction between time × treatment influenced the concentration of all analytes (*p* < 0.001). Treatment had the greatest effect on furan compounds (2-acetylfuran, furfural, 5-methylfurfural, homofuraneol, furfuryl ethyl ether, and 2,3-dihydrobenzofuran; η^2^ > 0.5).

After 4 weeks of accelerated aging, MRP concentrations were generally lowest in control and fructose-only treatments, demonstrating that supplementary amino acid additions successfully enhanced MR activity. However, select MRPs were lower in fructose-cysteine treatments, as discussed below.

##### Fructose-Cysteine Treatments

2,3,5-trimethylpyrazine, homofuraneol, and furfural were found at lower levels in fructose-cysteine treatments compared to control or fructose-only conditions. Further, other MRPs, including 5-methylfurfural and 2,3-dihydrobenzofuran, were measured at similar levels in control, fructose-only, and fructose-cysteine wines. The reduced concentration of these MRPs in fructose-cysteine conditions was evident at the 2- or 4-week interval, indicating that cysteine is not involved in their formation and/or may inhibit their proliferation by consuming reactant species to produce thiazoles and other sulfur-containing volatiles [5,73]. In contrast, fructose-cysteine systems had the highest average concentrations of benzaldehyde, ethyl-2-furoate, and thiazole at 4 weeks of aging (Supplementary Table S10). While mean benzaldehyde levels were only marginally higher in fructose-cysteine conditions, ethyl-2-furoate and thiazole were approximately 3- and 7-fold higher, respectively. The chemical composition of benzaldehyde and ethyl-2-furoate shows no apparent links to the structure of cysteine, and the reason for their increase is presently unknown. The large increase in thiazole, an *N*,*S*-heterocycle, is a product of Maillard activity between cysteine and carbonyls in wine [9,74]. However, thiazole concentrations were below its ODT of 38 mg/L [9].

##### Fructose-Lysine Treatments

At 4 weeks, fructose-lysine treatments contained the highest levels of furfural, 5-methylfurfural, homofuraneol, and 2,3-dihydrobenzofuran. Elevated levels of these furan intermediate compounds likely relate to elevated browning measured in fructose-lysine treatments during aging [75]. Furfural levels were above the ODT of 14 mg/L at 2- and 4-week intervals for all treatments except those containing cysteine [76]. In fructose-lysine treatments, furfural had an OAV of 17.7, indicating that fruity, caramel, and toasted aroma qualities were likely pronounced [76], although sensory evaluation was not conducted. Similarly, the 1 mg/L ODT for 5-methylfurfural was exceeded in all samples after 2 and 4 weeks of aging, with a maximum OAV of 30.6 in fructose-lysine treatments [77]. 5-Methylfurfural contributes sweet, caramel, nutty, and spicy characteristics to wine [76,77]. Homofuraneol exceeded its ODT of 10 mg/L in conditions containing glycine and lysine [78], with a maximum OAV of approximately 1.3 in fructose-lysine treatments and aroma qualities of strawberry and caramel [79]. No ODT has been reported for 2,3-dihydrobenzofuran. The elevated concentration of many furan compounds in model wines containing lysine is consistent with the high reactivity of lysine in Maillard systems [18,48]. However, a previous study reported conversely that the presence of lysine inhibited the formation of furan compounds in model Maillard systems when heated to 180 °C in combination with glucose (no pH information provided) [80]. The lower temperature of our reaction conditions (50 °C) and the presence of fructose rather than glucose, as well as other matrix-specific factors may account for this discrepancy, emphasizing the importance of comparing reaction conditions when evaluating MRP formation in different model systems.

##### Fructose-Glycine Treatments

As previously discussed, fructose-glycine treatments contained the highest level of 2,3,5-trimethylpyrazine after 4 weeks of aging (*p* < 0.05), and this MRP is a known product of Maillard pathways involving glycine [71]. Concentrations were approximately 3-fold higher in treatments containing glycine compared to those with cysteine (lowest overall 2,3,5-trimethylpyrazine levels) and were an average of 1.3-fold higher compared to all other treatments. To our knowledge, no ODT has been reported for 2,3,5-trimethylpyrazine. Glycine is a simple amino acid with a side chain of a single hydrogen atom, and as such, it has been commonly used as a model reagent for studying the MR [38,43,81,82]. Glycine is known to be involved in the formation of various pyrazines, including 2,3,5-trimethylpyrazine, in a pH-dependent manner [71]. The formation of pyrazines from glycine is generally favored by a high pH. However, many pyrazines, including 2,3,5-trimethylpyrazine, 2,5-dimethylpyrazine, 2,3-diethylpyrazine, 2-ethyl-3,5-dimethylpyrazine, 2,3,5,6-tetramethylpyrazine, 2,3-diethyl-5-methylpyraine, quinoxaline, and 2-methylquinoxaline, have been previously reported to form at pH 2.34 in glucose–glycine model systems (120 °C for 5 h) [71], which is relevant to sparkling wine (pH ~3). An expanded investigation of Maillard-derived pyrazine compounds would be beneficial in examining their prevalence in sparkling wines under typical cellar aging conditions and their sensory implications.

#### 2.5.3. Influence of Ca and Mg

Metals were a contributor to the variation in concentration for benzaldehyde, homofuraneol, furfuryl ethyl ether, and 2,3,5-trimethylpyrazine (*p* < 0.05), although discrete trends were only identified in Ca treatments for benzaldehyde and furfuryl ethyl ether. The effect of all metal-related contributions and their interactions was generally small (η^2^ < 0.01), apart from a moderate effect for furfuryl ethyl ether in relation to the metal × treatment interaction (*p* < 0.05; η^2^ = 0.06).

At 4 weeks, high levels (50 mg/L) of Ca^2+^ were associated with elevated concentrations of benzaldehyde and furfuryl ethyl ether compared to wines without metal additions (Supplementary Table S11). Benzaldehyde levels were approximately 3 mg/L higher in fructose-cysteine treatments with high Ca^2+^ after 4 weeks, whereas furfuryl ethyl ether was 1 mg/L higher in these conditions. Despite the small concentration increases in these treatments, potential synergistic effects require further study to determine their sensory relevance.

No trends were observed for Mg treatments, indicating that Mg plays a lesser role in Maillard-related interactions under wine conditions.

Although further research is needed, our results provide evidence that elevated Ca content may be related to higher levels of select MRPs in aged wines. The source of Ca in wine is primarily derived from the grape skin and pulp. However, this can be influenced by soil Ca levels, grape maturity, variety, climatic conditions, cement winemaking vessels, and adjuvants during winemaking [83]. Ca levels above 70–80 mg/L are considered at risk of instability, leading to Ca tartrate precipitation, although other factors, including malic acid content and pH, can influence precipitation and stability [83,84]. Regions with naturally Ca-rich soils and winemaking practices that introduce Ca to a final level below 70–80 mg/L may benefit from positive wine age-ability due to MRP formation during storage.

#### 2.5.4. Limitations of the Study and Areas of Future Research

A limitation of this study is the absence of a hydroalcoholic model base wine in the design to eliminate matrix effects derived from the wine matrix. Instead, sparkling base wine was modified by supplementing select chemical components (i.e., divalent metal ions, sugar, amino acids) prior to accelerated aging. Chardonnay base wine from the 2019 vintage was used as the base wine in all treatments and was stored at 4 °C for approximately three years prior to its use in this experiment, with free SO_2_ levels maintained at 20 ppm. These conditions were intended to reduce MRP formation during storage. The justification for selecting a base wine rather than a hydroalcoholic model wine is two-fold: (1) the MR is a highly complex reaction cascade and interactions between discrete reactant species in the absence of matrix effects may not sufficiently represent reaction pathways and products in wine, and (2) sparkling winemakers often utilize and/or blend base wines from past vintages to create a reserve base wine, which undergoes aging prior to bottling for the second alcoholic fermentation. By utilizing a base wine from a past vintage, experimental conditions and outcomes have relevance to current industry practices.

As previously mentioned, accelerated aging by mild heating remains a convenient approach to analyze the chemical evolution of wine over a reduced time frame compared to conventional aging; however, the composition of MRPs has been reported to diverge according to reaction temperature [10,37,38,39]. Due to this limitation, the outcomes from this study highlight areas for future research on MR-associated compound development in base wines; specifically, comparisons between accelerated and conventional aging regimes. In addition, this future research may be able to determine a reference treatment duration for accelerated aging which parallels key 6-, 12-, 18-, and 24-month aging periods in conventional cellar aging.

The sensory implications of MRPs in accelerated and conventionally aged sparkling base wine and finished wine are also important gaps in this field of research that require further research. In the present study, low OAVs were measured for many MRPs, although synergistic and additive effects of sub- and peri-threshold odorants may contribute to an overall perceptible impact on sensory qualities [85] and warrant further investigation.

## 3. Materials and Methods

### 3.1. Chemicals and Standards

Potassium metabisulfite (KMS) and potassium bitartrate (cream of tartar) were purchased from Vines to Vintages (Jordan, ON, Canada). Zymaflore^®^ Spark yeast and diammonium phosphate (DAP) were purchased from Laffort (Bordeaux, France). Go-Ferm^®^ yeast nutrient was purchased from Lallemand (Montreal, QC, Canada). d-Fructose (Fru; >99.5%) was purchased from BioShop Canada Inc. (Burlington, ON, Canada). Glycine (Gly; >99%), l-lysine (Lys; >98%), cysteine (Cys; 97%), calcium chloride (CaCl_2_; >97%), and magnesium chloride (MgCl_2_; >98%) were purchased from Sigma-Aldrich (Oakville, ON, Canada).

A composite solution of 40 mmol/L benzimidazole and 10 mmol/L tartaric acid as well as a solution of 10 mmol/L 18-crown-6 were purchased from Lumex Analytics (Wakendorf II, Germany). Sodium phosphate dibasic dodecahydrate (CAS 10039-32-4; ≥99%), sodium phosphate monobasic dihydrate (CAS 13472-35-0; ≥99%), sodium carbonate decahydrate (CAS 6132-02-1; ≥99%), and phenyl isothiocyanate (PITC; CAS 103-72-0; ≥99%) were purchased from Sigma-Aldrich (Oakville, ON, Canada). b-Cyclodextrin (CAS 7585-39-9; >99%) was purchased from TCI America (Portland, OR, USA). Isopropyl alcohol (>99%) was purchased from Fisher Chemical (Fair Lawn, NJ, USA). Concentrated hydrochloric acid (HCl; ≥37%) was purchased from Sigma-Aldrich (Oakville, ON, Canada). Sodium hydroxide (NaOH; ≥1.0 N in aqueous solution) was purchased from VWR International (Radnor, PA, USA). Nitrogen gas (99.665%) was purchased from Linde Canada Inc. (Mississauga, ON, Canada).

Chemical standards for the HS-SPME-GC/MS method were obtained from Sigma Aldrich (St. Louis, MO, USA), Fisher Scientific (Mississauga, ON, Canada), and Tokyo Chemical Industry (Tokyo, Japan), and internal standards from CDN Isotopes (Pointe-Claire, QC, Canada) according to Charnock et al. (2023) [67]. Milli-Q water was obtained from Biocel water purifying system (Millipore, Etobicoke, ON, Canada) and filtered through a 0.22 µm filter (Millipore). Sodium chloride (NaCl; ≥99%) was purchased from Fisher Chemical (Fair Lawn, NJ, USA). Absolute anhydrous ethanol was purchased from Greenfield Global (Mississauga, ON, Canada). GC-MS grade methanol (≥99.8%) was purchased from Millipore Sigma (Burlington, VT, USA).

### 3.2. Primary Fermentation and Winemaking

Treatments were prepared in a matrix of Vitis vinifera cv. Chardonnay base wine produced from the 2019 vintage. Grapes were hand harvested on 19 September 2019 from Trius Winery in Niagara-on-the-Lake, ON, Canada, and transported to the Cool Climate Oenology & Viticulture Institute (CCOVI) Research Winery. Primary fermentation was carried out with the commercial oenological S. cerevisiae strain, Zymaflore^®^ Spark (Laffort, Bordeaux, France). The yeast was rehydrated according to manufacturer guidelines and starter cultures were built up in sterile-filtered Chardonnay must from the same harvest. Go-Ferm^®^ yeast nutrient (Lallemand, Montreal, QC, Canada) was added to the rehydration preparation at 0.3 g/L. Yeast assimilable nitrogen (YAN) was adjusted to 25 mg/L by the addition of diammonium phosphate (DAP; Laffort, Bordeaux, France).

Following inoculation, fermentations were gently mixed and moved to a temperature-controlled chamber at 16 °C where they were monitored once daily for total soluble solids by hydrometer (°Brix) and temperature (°C). Fermentations were considered completed when the residual sugar concentration reached dryness (<5 g/L) and was consistent for three consecutive days. Wines were then racked off their lees and 60 ppm of sulfur dioxide (SO_2_) was added as potassium metabisulfite (KMS; Vines to Vintages, Jordan, ON, Canada). Subsequently, wines were blanketed with carbon dioxide (CO_2_) and stored at 4 °C for approximately three years, during which time SO_2_ levels were routinely analyzed and maintained at 20 ppm free SO_2_. In October 2022, the base wine was cold stabilized at −2 °C for ten days following the addition of potassium bitartrate (Vines to Vintages, Jordan, ON, Canada) at the manufacturer recommended dose rate of 4 g/L. Afterward, the wine was racked off and coarse filtered using a 5–7 mm filter pads (#1 coarse filter pads, Buon Vino, Cambridge, ON, Canada) with a benchtop filtration unit (Super Jet, Buon Vino, Cambridge, ON, Canada).

### 3.3. Standard Wine Chemical Analysis

Measurements for pH and titratable acidity (TA) were obtained using a Hanna Instruments HI 84502 auto-titrator (Woonsocket, RI, USA) which was calibrated with standard solutions of pH 4.0, 7.2, and 10.0. To ensure accurate auto-titration, a pump calibration standard (HI 84502-55, Hanna Instruments, Woonsocket, RI, USA) was employed. Ethanol content (% *v*/*v*) was analyzed by gas chromatography-flame ionization detection (GC-FID) using a modified method from Nurgel et al. (2004) [86], with adaptations including the use of an Agilent 6890 GC-FID (Agilent, Santa Clara, CA, USA) equipped with a DB wax column (30 m × 0.25 mm × 0.25 µm), an Agilent 7638B automated split/spitless injector (Agilent, Santa Clara, CA, USA), and an internal standard of 0.1% butanol. The aspiration method outlined by Iland et al. (2015) was used to analyze free and total SO_2_ levels [87]. The degree of browning was measured as the absorbance at λ_420nm_ (A_420_) in a 1 cm spectrophotometric cell with a Cary 60 UV-Vis spectrophotometer (Agilent, Santa Clara, CA, USA), and the results were multiplied by 1000 (mAU). Megazyme^®^ enzymatic assay kits were used to measure sugar and organic acid composition (K-FRUGL, L-MAL, K-ACET; Bray, Ireland). Prior to treatment additions, the base wine was analyzed for standard chemical parameters as shown in Table 3.

Prior to treatment additions, the base wine was profiled for total metal composition by inductively coupled plasma mass spectrometry (ICP-MS) [26]. Metal analysis was carried out by the Queen’s University Analytical Services Unit. The base wine was analyzed in triplicate and metal levels had a mean relative standard deviation of 4.2 ± 2.1%. Results are shown in Supplementary Table S12. Ca and Mg (± SD) were measured at 62 ± 4 and 60 ± 2 mg/L, respectively, in the base wine.

### 3.4. Modified Base Wine Model Systems

Model conditions were prepared according to Table 4. Single sugar–amino acid combinations were prepared to 0.02 M fructose (2 molar equivalents) and to 0.01 M (1 molar equivalent) of the applicable amino acid (Gly, Lys, Cys). Additional treatments included a control (no sugar or amino acid) and fructose-only conditions.

Bulk preparations of each treatment group were prepared in 2 L Schott bottles with screw top lids (Fisherbrand, Saint-Laurent, QC, Canada) to homogenize amino acid and sugar additions. Individual reactions (200 mL) were aliquoted from the bulk solution and transferred to separate 500 mL Schott bottles with screw top lids and prepared in duplicate. Bottles were pre-washed with a 16 h soak (Sparkleen laboratory detergent, Fisher Scientific, Pittsburgh, PA, USA) and copious rinsing with Milli-Q water prior to use [34].

Selective additions of Ca^2+^ and Mg^2+^ at low and high concentrations (10 and 50 mg/L, respectively) were carried out by spiking 0.4 mL of concentrated stock solutions into individual 200 mL treatment volumes. This protocol follows a similar experimental design to Viviers et al. (2013) wherein the spike is intended to add negligible volume to the overall sample. High and low stock solutions (25,000 mg/L and 5000 mg/L, respectively) were prepared for each metal by dissolving CaCl_2_ or MgCl_2_ in deionized water [88].

Initial (0-week) samples were collected upon stirring each bottle on a stir plate while an approximately 50 mL sample was extracted (leaving 150 mL per bottle). The headspace was blanketed with CO_2,_ and samples were transferred to a Puffer-Hubbard Uni-Therm 800 Series incubator cabinet with a programmed Honeywell temperature control unit (Thermo Fisher Scientific, Waltham, MA, USA) at 50 °C for 4 weeks, with subsequent sampling intervals at 2- and 4-week intervals. At each sampling interval, 50 mL of wine was collected per vessel and samples were heavily blanketed with CO_2_. Collected samples were partitioned into a 20 mL aliquot for immediate chemical analysis, and a 30 mL aliquot was frozen as a sample archive. Analysis at each interval included standard wine chemical parameters of pH, TA, and A_420_, determination of sugar levels by enzymatic assay, free Ca^2+^ and Mg^2+^ by capillary electrophoresis (CE), select free amino acid monitoring by CE, and the analysis of MRPs by headspace solid-phase microextraction coupled with gas chromatography–mass spectrometry (HS-SPME-GC/MS).

### 3.5. Determination of Metals and Free Amino Acids by CE

The quantification of selected metal cations and free amino acids was carried out separately by electrophoretic techniques using previous methodology [89,90], with details described in subsequent sections. Both analyses utilized the Capel 105-M system (Lumex, St. Petersburg, Russia) equipped with a UV detector. Instrument control, signal acquisition, and analyte determination were performed by Elforun^®^ software version 3.2.5. Target analytes were identified according to their retention times and quantified by peak integrations with linear calibration curves. A sample electropherogram is shown in Supplementary Figure S1. Analyses were carried out in duplicate.

#### 3.5.1. Magnesium and Calcium Analysis

##### Instrumental Parameters

Select metals of interest in the model treatments (i.e., Ca and Mg) were measured indirectly [89], with protocol information made available by the Lumex method for cations in beverages (Method ID 0300002735). A capillary cassette containing a fused silica capillary of 60 cm total length × 75 mm inner diameter (50 cm effective length; Lumex, St. Petersburg, Russia) was employed, and UV detection was carried out at 267 nm. The thermostating liquid was held at 20 °C during analysis. Prior to use, the capillary was conditioned for 5 min with MilliQ water, 30 min with 0.5 M HCl, 30 min with MilliQ water, 30 min with 0.5 M NaOH, and again for 30 min with MilliQ water, with each rinse under 1000 mbar pressure. Between each sample run, the capillary was rinsed for 3 min with water, then for 5 min with 0.5 M NaOH, 5 min with water, and 3 min with the background electrolyte buffer solution. The injection of analytes into the capillary was performed under pressure (30 mbar, 5 s) and analyzed under a voltage of +25 kV.

##### Solution and Sample Preparation

Individual stock solutions of Ca and Mg were prepared to 1 mg/L in MilliQ water. A composite standard solution was then prepared to contain 50 mg/L Ca and 25 mg/L Mg and diluted in MilliQ water. Subsequent standard solutions were prepared by dilution for a total of five calibration standards per analyte with a linear range of 0.50–50 mg/L for Ca and 0.25–25 mg/L for Mg, with R^2^ values > 0.99 (Supplementary Figure S2). MilliQ water was tested as a blank solution to ensure samples and standards were free of cation impurities. Coefficients of variation were <9%.

The run buffer (background electrolyte solution; BGE) for metals analysis contained 20 mmol/L of benzimidazole, 5 mmol/L of tartaric acid, and 2 mmol/L of 18-crown-6. This solution was filtered through a 0.45 mm syringe filter (Fisher Scientific, Mississauga, ON, Canada) prior to use to prevent capillary obstruction. Prior to analysis, all wine samples were also filtered through a 0.45 mm syringe filter, diluted 10-fold (*v*/*v*) in MilliQ water, and centrifuged at 5000 rpm for 5 min. Reported concentrations represent final levels in the treatments without dilution.

#### 3.5.2. Free Amino Acid Analysis

##### Instrumental Parameters

Free amino acids used in treatment additions (Gly, Lys, Cys) were determined by CE in the form of phenyl isothiocyanate derivatives (PITC-derivatives) [90]. Only free amino acids were analyzed. A cassette containing a fused silica capillary of 75 cm total length × 50 mm inner diameter (65 cm effective length; Lumex, St. Petersburg, Russia) was used, UV detection was performed at 254 nm, and the thermostating temperature was held at 30 °C. Capillary conditioning prior to analysis and rinsing between sample runs followed the regime previously outlined for metals analysis in Section 3.5.1 “Instrumental Parameters”. The injection of analytes was performed under pressure (30 mbar, 5 s) and with a voltage of +25 kV.

##### Solution and Sample Preparation

The BGE buffer solution was prepared according to previous methods [90]. The phosphate component of the BGE was initially prepared as two individual solutions: one of 134 mmol/L sodium phosphate dibasic dodecahydrate and a separate preparation of 165 mmol/L sodium phosphate monobasic dihydrate. These two solutions were combined to generate a composite phosphate buffer of pH 7.7–7.8 containing 75 mmol/L phosphate ion (PO_4_^3−^). A 10 mL aliquot of the composite buffer was then mixed with 10 mL of a 10 mmol/L b-cyclodextrin solution and diluted to 25 mL, yielding a final BGE buffer solution which contained 4 mmol/L b-cyclodextrin and 30 mmol/L phosphate buffer. All solutions were prepared in MilliQ water.

Stock solutions for Gly, Lys, and Cys were individually prepared to 4.0 g/L in MilliQ water. Cysteine required pre-treatment by dissolution in a minimal amount of 1.0 M HCl (generating cysteine hydrochloride) to prevent oxidation and precipitation, ensuring its solubility in an aqueous solution. A composite standard of Gly, Lys, and Cys containing 100 mg/L of each amino acid was used to prepare five calibration standards. Prior to analysis, free amino acids were derivatized with phenyl isothiocyanate (PITC) to facilitate their detection and quantification. Without derivatization, amino acids lack strong chromophores that are necessary for their detection by UV. PITC (also called Edman’s reagent) reacts with the amino groups of amino acids to generate stable phenylthiocarbamyl (PTC) derivatives, which are quantified by measuring their UV absorbance and corresponding peak area, which is directly proportional to concentration [91]. For calibration standards, individual aliquots of the composite standard solution were evaporated under a stream of N_2_ to dryness. Each residue was subsequently dissolved in 150 mL of a 0.1 mol/L sodium carbonate solution (prepared in MilliQ water) and 300 mL of a 0.1 mol/L PITC solution (prepared in isopropyl alcohol) and stirred until precipitate dissolution. Samples were held in sealed vials at ambient temperature for 35 min, followed by subsequent evaporation under N_2_ to dryness. The derivatized residue was then dissolved in 1 mL MilliQ water and centrifuged at 5000 rpm for 5 min prior to analysis. Calibration standards were prepared to a linear range of 5–60 mg/L with R^2^ values > 0.99 (Supplementary Figure S3). Measured coefficients of variation were <15%. For wine samples, 25 mL wine was evaporated to dryness prior to derivatization and analysis (40-fold *v*/*v* dilution).

#### 3.5.3. Maillard Reaction-Associated Product Determination by HS-SPME-GC/MS

HS-SPME-GC/MS was applied for the determination of MRPs, according to Charnock et al. 2023 [67]. Briefly, analysis was carried out using an Agilent 7890B GC and 5977B quadrupole MSD (Santa Clara, CA, USA) equipped with a DB-624UI capillary column (30 m × 0.25 mm, 1.4 µm film thickness, Agilent Technologies, Santa Clara, CA, USA) and PAL RSI 85 autosampler (CTC Analytics, Zwingen, Switzerland). The autosampler was outfitted with a Peltier stack tray cooler, which held samples at 4 °C until analysis (model G4565A, CTC Analytics, Zwingen, Switzerland). An 85 µm Carboxen/polydimethylsiloxane (CAR/PDMS) coated 23-gauge metal alloy SPME fiber (Supelco^®^, Bellefonte, PA, USA) was utilized for headspace analysis. Instrumental parameters included vial agitation at 250 rpm for 5 min at 40 °C before inserting the fiber into the headspace for 55 min at 40 °C throughout continued agitation (250 rpm). The sample was desorbed from the fiber to the inlet at 250 °C for 5 min. Helium carrier gas (Ultra-high purity 5.0, Linde Canada Inc., Mississauga, ON, Canada) at a flow rate of 1.0 mL/min was used, with the oven program as follows: the initial temperature was 40 °C for 4 min, ramped at 2 °C/min to 160 °C, held for 1 min, ramped at 5 °C/min to 230 °C, and held for 5 min. All analysis was carried out in selective-ion monitoring (SIM) mode, and data were processed with Agilent OpenLab software (2.4.5.9, Agilent Technologies).

Six calibration standards were prepared over a range of 1–100 mg/L for all analytes except furfural, which included a seventh standard at 300 mg/L. A composite internal standard containing benzaldehyde-d_6_, furfural-d_4_, and 2-methylpyrazine-d_6_ was added to a final concentration of 20 µg/L. Acceptable regression coefficients were determined for all analytes (R^2^ > 0.9). Samples (4.9 mL) were prepared with 1.5 g NaCl and 0.1 mL composite internal standard in a 10 mL amber glass vial for analysis. Vials were immediately sealed by screw cap and stored at 4 °C for <24 h until analysis. Analyses were carried out on duplicate treatment preparations with analytical duplicates and coefficients of variation <14%.

Compound identification was carried out by comparing retention times and mass spectra to pure compounds using the NIST spectral database. Standard concentrations were quantified based on the ratio of the peak area of the compound to the corresponding deuterated internal standard. Odor activity values (OAVs) were determined by dividing the mean concentration of the compound by its reported odor detection threshold (ODT).

#### 3.5.4. Statistical Analysis

Statistical analysis was carried out with XLSTAT Version 2022.4.1 (Addinsoft Inc., New York, NY, USA). Standard wine chemical analysis, sugars, and MRPs were analyzed by three-way Analysis of Variance (ANOVA) with Tukey’s Honest Significant Difference (HSD) tests to evaluate the effect of fructose and amino acid combination (treatment), metal addition (metal), accelerated aging duration (time) factors, and their interactions. Free amino acids glycine, lysine, and cysteine were assessed in their respective model systems by a two-way ANOVA with Tukey’s HSD tests to evaluate the effect of time, metal addition, and their interaction. Significance was established at *p* < 0.05. Eta-squared (η^2^) values derived from the three-way ANOVA with all interactions included in the model used to assess the relative effect size of each factor (small, >0.01; medium, >0.06; large, >0.140) [92].

## 4. Conclusions

The results from the present study confirmed that the MR proceeds under wine pH, leading to the formation of various intermediate reaction products, which are influenced by the composition of reactant species as well as, to a much lesser extent, the presence of metal ions. By means of accelerated aging conditions with mild heating, we identified the selective formation and inhibition of reaction products according to the amino acid present in the matrix and, moreover, the heightened reactivity of lysine in relation to furan-derived compound formation and browning. Further, the presence of cysteine inhibited the formation of many compounds to levels below those measured in the control or fructose-only conditions, although its presence also elevated levels of thiazole and ethyl-2-furoate. The presence of 50 mg/L Ca was associated with slightly elevated concentrations of benzaldehyde and furfuryl ethyl ether when in the presence of cysteine, although the sensory relevance of this difference was not assessed. While few MRPs were found at concentrations above their odor threshold, it is hypothesized that these compounds, among others not included in this targeted study, may act synergistically to contribute “aged” aromas to the wine. The influence of elevated Ca^2+^ on a broader range of MRPs during cellar aging of reserve base wines and sparkling wines requires further research.

## Figures and Tables

**Table 1:** Concentration of Maillard reaction-associated products (mg/L) during accelerated aging (at 50 °C) of modified Chardonnay base wine model systems for 4 weeks, evaluated by a three-way ANOVA with Tukey’s post hoc test.

Model System	Reaction Time (Weeks)											
	0	2	4	0	2	4	0	2	4	0	2	4
	Benzaldehyde			2-Acetylfuran			Furfural			5-Methylfurfural		
CT	**4.2 ± 0.2**	**7.1 ± 0.9**	**11.0 ± 0.8**	n.q.	1.9 ± 0.2	4.6 ± 0.5	n.d.	**48.0 ± 1.9**	**98.0 ± 3.8**	n.q.	**4.1 ± 0.2**	**6.8 ± 0.4**
CT ML		**7.0 ± 0.6**	**11.3 ± 0.4**		2.0 ± 0.2	4.5 ± 0.6		**45.8 ± 2.1**	**101.1 ± 8.3**		**4.1 ± 0.1**	**7.2 ± 0.4**
CT MH		**7.7 ± 0.7**	**11.7 ± 0.5**		2.3 ± 0.1	4.4 ± 0.4		**46.0 ± 2.2**	**102.5 ± 8.4**		**4.1 ± 0.1**	**7.5 ± 0.4**
CT CL		**8.3 ± 0.8**	**12.1 ± 0.5**		2.2 ± 0.1	4.4 ± 0.4		**42.3 ± 1.6**	**102.6 ± 8.8**		**4.0 ± 0.2**	**6.9 ± 0.4**
CT CH		**8.5 ± 0.8**	**12.0 ± 0.5**		2.0 ± 0.2	4.2 ± 0.3		**37.2 ± 1.4**	**98.4 ± 10.7**		**3.8 ± 0.2**	**7.0 ± 0.4**
F	**4.4 ± 0.2**	**7.6 ± 0.6**	**12.1 ± 0.6**	n.q.	2.2 ± 0.1	4.5 ± 0.3	n.d.	**47.6 ± 1.9**	**99.4 ± 12.1**	n.q.	**4.1 ± 0.1**	**7.1 ± 0.4**
F ML		**8.4 ± 0.9**	**12.5 ± 0.5**		2.3 ± 0.2	4.7 ± 0.5		**46.1 ± 1.6**	**99.5 ± 10.7**		**4.0 ± 0.2**	**7.1 ± 0.4**
F MH		**8.2 ± 0.6**	**12.4 ± 0.5**		2.3 ± 0.1	4.4 ± 0.4		**45.6 ± 1.5**	**100.8 ± 10.4**		**4.1 ± 0.2**	**7.0 ± 0.2**
F CL		**8.6 ± 0.6**	**11.9 ± 0.5**		2.2 ± 0.1	4.7 ± 0.4		**42.3 ± 1.3**	**99.4 ± 8.3**		**4.0 ± 0.1**	**7.1 ± 0.5**
F CH		**8.6 ± 0.8**	**12.3 ± 0.4**		2.0 ± 0.2	5.0 ± 0.5		**36.3 ± 2.3**	**101.2 ± 8.4**		**3.8 ± 0.2**	**7.1 ± 0.6**
FG	**4.6 ± 0.2**	**8.4 ± 0.2**	**13.7 ± 0.4**	n.q.	6.8 ± 0.5	17.9 ± 2.3	n.d.	**207.0 ± 2.0**	**192.7 ± 19.4**	n.q.	**10.2 ± 0.7**	**25.3 ± 2.0**
FG ML		**8.3 ± 0.6**	**13.6 ± 1.1**		7.6 ± 0.1	16.8 ± 1.6		**223.3 ± 6.4**	**214.6 ± 31.0**		**10.8 ± 0.4**	**25.6 ± 1.8**
FG MH		**8.6 ± 0.9**	**12.8 ± 0.3**		7.5 ± 0.5	18.5 ± 1.8		**228.3 ± 6.6**	**201.2 ± 20.8**		**11.7 ± 0.8**	**26.7 ± 1.8**
FG CL		**8.7 ± 1.1**	**12.9 ± 0.6**		7.6 ± 0.2	16.8 ± 1.4		**222.7 ± 1.8**	**199.5 ± 17.6**		**11.2 ± 0.6**	**27.0 ± 0.9**
FG CH		**8.8 ± 0.9**	**13.4 ± 0.3**		7.5 ± 0.5	16.8 ± 1.5		**225.0 ± 3.4**	**205.6 ± 28.4**		**11.2 ± 0.5**	**26.0 ± 2.0**
FL	**4.6 ± 0.1**	**9.8 ± 0.8**	**13.8 ± 0.5**	n.q.	6.8 ± 0.5	16.6 ± 1.6	n.d.	**279.8 ± 5.2**	**251.2 ± 17.9**	n.q.	**12.2 ± 0.8**	**31.1 ± 2.0**
FL ML		**9.4 ± 0.5**	**13.4 ± 0.8**		6.4 ± 0.4	17.9 ± 1.7		**273.1 ± 4.1**	**235.8 ± 18.3**		**11.8 ± 0.6**	**31.4 ± 2.3**
FL MH		**9.1 ± 0.7**	**12.8 ± 0.6**		6.6 ± 0.3	16.5 ± 1.6		**280.1 ± 6.7**	**244.8 ± 27.3**		**11.5 ± 0.6**	**30.8 ± 1.9**
FL CL		**9.2 ± 0.3**	**13.9 ± 0.4**		6.7 ± 0.3	15.8 ± 1.7		**282.4 ± 7.8**	**251.1 ± 21.7**		**12.0 ± 0.7**	**29.2 ± 0.7**
FL CH		**9.1 ± 0.7**	**14.0 ± 0.7**		6.5 ± 0.4	15.2 ± 1.2		**270.0 ± 1.7**	**258.8 ± 32.2**		**11.1 ± 0.5**	**30.4 ± 1.6**
FC	**3.7 ± 0.2**	**7.7 ± 0.6**	**13.9 ± 0.5**	n.q.	5.6 ± 0.3	12.0 ± 0.8	n.d.	n.d.	4.4 ± 0.5	n.q.	**5.7 ± 0.3**	**8.5 ± 0.4**
FC ML		**8.2 ± 0.7**	**14.3 ± 1.2**		5.6 ± 0.4	11.4 ± 0.8		n.d.	4.6 ± 0.4		**5.3 ± 0.2**	**8.6 ± 0.4**
FC MH		**8.7 ± 0.6**	**13.5 ± 0.9**		5.3 ± 0.4	11.2 ± 0.9		n.d.	5.7 ± 0.4		**5.3 ± 0.2**	**8.5 ± 0.8**
FC CL		**8.3 ± 1.0**	**13.5 ± 1.1**		5.6 ± 0.6	11.5 ± 0.7		n.d.	n.d.		**5.3 ± 0.2**	**8.6 ± 0.3**
FC CH		**10.7 ± 0.9**	**17.5 ± 1.5**		6.0 ± 0.4	11.7 ± 1.2		n.d.	n.d.		**5.3 ± 0.2**	**8.5 ± 0.3**
Time	A	B	C	A	B	C	A	B	C	A	B	C
	Homofuraneol			Furfuryl ethyl ether			Ethyl-2-furoate *			2,3-Dihydrobenzofuran *		
CT	n.q.	2.5 ± 0.0	3.6 ± 0.1	n.q.	1.9 ± 0.1	2.0 ± 0.1	4.6 ± 0.1	11.6 ± 0.4	23.2 ± 1.3	n.q.	4.1 ± 0.2	6.8 ± 0.4
CT ML		2.4 ± 0.1	3.6 ± 0.0		1.9 ± 0.1	2.1 ± 0.2		11.7 ± 0.4	23.8 ± 0.9		4.1 ± 0.1	7.2 ± 0.4
CT MH		2.3 ± 0.0	3.7 ± 0.1		2.0 ± 0.2	2.1 ± 0.2		12.2 ± 0.2	20.1 ± 1.2		4.1 ± 0.1	7.5 ± 0.4
CT CL		2.3 ± 0.1	3.5 ± 0.1		2.0 ± 0.2	2.1 ± 0.2		11.9 ± 0.2	21.0 ± 1.7		4.0 ± 0.2	6.9 ± 0.4
CT CH		2.3 ± 0.0	3.5 ± 0.1		1.7 ± 0.2	2.1 ± 0.2		11.0 ± 0.6	20.6 ± 1.9		3.8 ± 0.2	7.0 ± 0.4
F	n.q.	2.4 ± 0.1	3.5 ± 0.1	n.q.	2.0 ± 0.1	2.1 ± 0.2	4.8 ± 0.1	11.8 ± 0.6	20.5 ± 1.7	n.q.	4.1 ± 0.1	7.1 ± 0.4
F ML		2.4 ± 0.1	3.5 ± 0.1		1.9 ± 0.1	2.1 ± 0.2		11.9 ± 0.6	20.5 ± 1.8		4.0 ± 0.2	7.1 ± 0.4
F MH		2.3 ± 0.1	3.6 ± 0.1		1.9 ± 0.1	2.2 ± 0.2		11.9 ± 0.7	20.8 ± 1.9		4.1 ± 0.2	7.0 ± 0.2
F CL		2.3 ± 0.1	3.5 ± 0.1		2.0 ± 0.1	2.1 ± 0.2		11.7 ± 0.6	21.2 ± 1.4		4.0 ± 0.1	7.1 ± 0.5
F CH		2.2 ± 0.0	3.5 ± 0.2		1.9 ± 0.2	2.0 ± 0.2		11.0 ± 0.5	21.2 ± 1.8		3.8 ± 0.2	7.1 ± 0.6
FG	n.q.	5.9 ± 0.1	**10.2 ± 0.1**	n.q.	**2.8 ± 0.1**	**2.8 ± 0.3**	4.8 ± 0.1	21.4 ± 1.2	35.1 ± 1.2	n.q.	10.2 ± 0.7	25.3 ± 2.0
FG ML		6.3 ± 0.1	**10.3 ± 0.3**		**2.9 ± 0.1**	**2.5 ± 0.1**		22.3 ± 0.7	34.5 ± 1.5		10.8 ± 0.4	25.6 ± 1.8
FG MH		6.3 ± 0.1	**10.4 ± 0.1**		**2.9 ± 0.1**	**2.8 ± 0.3**		22.6 ± 0.8	34.4 ± 1.7		11.7 ± 0.8	26.7 ± 1.8
FG CL		6.2 ± 0.0	**10.4 ± 0.2**		**2.8 ± 0.1**	**3.0 ± 0.3**		22.3 ± 1.1	34.9 ± 2.0		11.2 ± 0.6	27.0 ± 0.9
FG CH		6.3 ± 0.1	**9.9 ± 0.2**		**2.9 ± 0.1**	**2.7 ± 0.2**		22.6 ± 1.1	35.1 ± 3.2		11.2 ± 0.5	26.0 ± 2.0
FL	n.q.	7.5 ± 0.1	**12.7 ± 0.4**	n.q.	**2.7 ± 0.1**	**2.8 ± 0.3**	5.0 ± 0.1	19.7 ± 1.0	32.6 ± 1.8	n.q.	12.2 ± 0.8	31.1 ± 2.0
FL ML		7.3 ± 0.1	**12.9 ± 0.5**		**2.7 ± 0.1**	**2.8 ± 0.2**		19.4 ± 1.3	32.3 ± 1.8		11.8 ± 0.6	31.4 ± 2.3
FL MH		7.5 ± 0.1	**12.9 ± 0.5**		**2.7 ± 0.0**	**2.8 ± 0.2**		20.1 ± 0.8	32.5 ± 1.3		11.5 ± 0.6	30.8 ± 1.9
FL CL		7.6 ± 0.1	**12.6 ± 0.6**		**2.7 ± 0.1**	**2.8 ± 0.2**		19.9 ± 1.1	31.9 ± 1.2		12.0 ± 0.7	29.2 ± 0.7
FL CH		7.3 ± 0.1	**12.5 ± 0.5**		**2.6 ± 0.1**	**2.7 ± 0.1**		19.5 ± 1.2	31.8 ± 1.6		11.1 ± 0.5	30.4 ± 1.6
FC	n.q.	n.q.	n.q.	n.q.	**3.0 ± 0.1**	**2.5 ± 0.1**	5.5 ± 0.1	33.5 ± 1.4	43.1 ± 1.4	n.q.	5.7 ± 0.3	8.5 ± 0.4
FC ML					**3.0 ± 0.1**	**2.6 ± 0.3**		33.8 ± 1.8	41.5 ± 1.5		5.3 ± 0.2	8.6 ± 0.4
FC MH					**2.9 ± 0.2**	**2.6 ± 0.3**		34.7 ± 2.1	43.0 ± 2.1		5.3 ± 0.2	8.5 ± 0.8
FC CL					**3.3 ± 0.3**	**2.8 ± 0.2**		34.0 ± 2.5	42.1 ± 2.3		5.3 ± 0.2	8.6 ± 0.3
FC CH					**3.2 ± 0.2**	**3.6 ± 0.4**		33.0 ± 1.9	41.8 ± 2.1		5.3 ± 0.2	8.5 ± 0.3
Time	A	B	C	A	B	B	A	B	C	A	B	C
	Thiazole			2,3,5-Trimethylpyrazine *								
CT	n.q.	n.q.	3.3 ± 0.4	4.6 ± 0.4	9.9 ± 0.5	12.8 ± 0.6						
CT ML		n.q.	2.5 ± 0.3		8.3 ± 0.8	12.9 ± 0.9						
CT MH		n.q.	3.0 ± 0.4		11.7 ± 1.2	13.7 ± 0.9						
CT CL		n.q.	2.7 ± 0.3		10.3 ± 0.8	13.4 ± 0.4						
CT CH		n.q.	2.4 ± 0.3		8.4 ± 0.8	13.4 ± 1.2						
F	n.q.	n.q.	2.5 ± 0.3	4.6 ± 0.2	9.9 ± 1.1	12.7 ± 1.2						
F ML		n.q.	2.5 ± 0.3		9.7 ± 0.8	11.8 ± 1.2						
F MH		n.q.	2.7 ± 0.3		10.0 ± 1.0	12.1 ± 0.9						
F CL		n.q.	2.6 ± 0.2		9.9 ± 0.9	11.9 ± 1.0						
F CH		n.q.	3.0 ± 0.3		10.2 ± 0.8	14.8 ± 1.1						
FG	n.q.	2.1 ± 0.1	3.4 ± 0.4	4.3 ± 0.3	17.8 ± 2.3	15.1 ± 1.3						
FG ML		2.2 ± 0.1	3.3 ± 0.3		16.7 ± 1.9	14.0 ± 1.2						
FG MH		2.1 ± 0.1	3.3 ± 0.3		17.4 ± 3.1	17.5 ± 1.3						
FG CL		2.2 ± 0.1	3.1 ± 0.2		18.1 ± 1.6	18.9 ± 1.9						
FG CH		2.2 ± 0.1	3.1 ± 0.2		17.5 ± 1.4	17.5 ± 1.5						
FL	n.q.	2.3 ± 0.2	3.5 ± 0.4	4.1 ± 0.1	13.4 ± 0.9	12.3 ± 1.9						
FL ML		2.4 ± 0.1	3.3 ± 0.3		14.0 ± 0.7	13.0 ± 0.9						
FL MH		2.4 ± 0.2	3.2 ± 0.2		13.3 ± 1.2	13.3 ± 0.9						
FL CL		2.5 ± 0.2	3.3 ± 0.5		12.1 ± 0.8	12.1 ± 1.4						
FL CH		2.4 ± 0.1	2.7 ± 0.2		13.2 ± 0.5	12.4 ± 0.3						
FC	n.q.	6.3 ± 0.7	18.8 ± 2.2	4.7 ± 0.1	5.9 ± 0.5	5.5 ± 0.4						
FC ML		6.7 ± 0.7	21.4 ± 0.9		5.8 ± 0.3	5.3 ± 0.6						
FC MH		6.9 ± 0.8	19.2 ± 2.3		5.6 ± 0.5	5.8 ± 0.7						
FC CL		6.4 ± 0.7	19.0 ± 1.6		5.9 ± 0.5	5.6 ± 0.6						
FC CH		5.2 ± 0.3	18.3 ± 1.4		5.7 ± 0.3	5.3 ± 0.5						
Time	A	B	C	A	B	C						

Data represent the mean value ± standard deviation of duplicate preparations of each treatment analyzed in duplicate. Model system treatments include control (CT), fructose (F); fructose-glycine (FG), fructose-lysine (FL), and fructose-cysteine (FC), with metal additions of low Mg (10 mg/L; ML), high Mg (50 mg/L; MH), low Ca (10 mg/L; CL), and high Ca (50 mg/L, CH). Values below limit of detection = n.d.; values below limit of quantification = n.q. Unique capital letters indicate different means for each time interval (each chemical parameter assessed separately). Statistical tests include values below the limit of detection and quantification where necessary. Bold values indicate OAV > 1. * Indicates compounds with no reported sensory detection threshold.

**Table 2:** Three-way ANOVA results for Maillard reaction-associated products during accelerated aging (at 50 °C) of modified sparkling base wine model systems for 4 weeks.

	Benzaldehyde	2-Acetyl-Furan	Furfural	5-Methyl- Furfural	Homo- Furaneol	Furfuryl Ethyl Ether	Ethyl-2- Furoate	2,3-Dihydro- Benzofuran	Thiazole	2,3,5- Trimethyl- Pyrazine
*p*-value										
Time	***	***	***	***	***	***	***	***	***	***
Metal	***	n.s.	n.s.	n.s.	***	*	n.s.	n.s.	n.s.	**
Txmt	***	***	***	***	***	***	***	***	***	***
Ti × M	***	n.s.	n.s.	n.s.	n.s.	*	n.s.	n.s.	n.s.	*
Ti × Tx	***	***	***	***	***	***	***	***	***	***
M × Tx	***	n.s.	n.s.	n.s.	n.s.	***	n.s.	n.s.	**	***
Ti × M × Tx	***	n.s.	n.s.	n.s.	n.s.	**	n.s.	n.s.	n.s.	***
F-statistic										
Time	5380.76	2589.59	70.34	4900.53	8295.08	0.30	11,228.25	4900.53	1499.79	2008.62
Metal	11.60	0.60	0.36	0.61	5.14	3.28	0.78	0.61	2.18	3.55
Txmt	49.55	1045.32	2510.63	3069.72	17,113.78	251.51	1558.62	3069.72	1789.62	491.83
Ti × M	4.16	1.35	0.98	0.33	1.01	2.47	1.17	0.33	1.54	2.53
Ti × Tx	24.93	195.77	140.09	774.28	1468.73	8.61	365.12	774.28	986.50	164.04
M × Tx	5.96	1.02	1.11	1.47	1.04	4.80	0.88	1.47	2.70	3.49
Ti × M × Tx	2.27	1.36	0.56	0.74	1.19	2.60	0.79	0.74	1.41	2.20
Eta-squared (η^2^)										
Time	0.925	0.334	0.006	0.239	0.100	0.000	0.704	0.239	0.053	0.523
Metal	0.004	0.000	0.000	0.000	0.000	0.010	0.000	0.000	0.001	0.002
Txmt	0.017	0.540	0.939	0.600	0.826	0.755	0.196	0.600	0.727	0.256
Ti × M	0.003	0.001	0.000	0.000	0.000	0.007	0.000	0.000	0.000	0.003
Ti × Tx	0.017	0.101	0.039	0.151	0.071	0.026	0.092	0.151	0.200	0.171
M × Tx	0.008	0.002	0.002	0.001	0.000	0.058	0.000	0.001	0.004	0.007
Ti × M × Tx	0.006	0.003	0.001	0.001	0.000	0.031	0.001	0.001	0.001	0.009

Significance: n.s. = *p* > 0.05; * = *p* < 0.05; ** = *p* < 0.01; *** = *p* < 0.001.

**Table 3:** Standard chemical analysis of Chardonnay base wine prior to treatment additions.

Chemical Parameter	Chardonnay Base Wine
pH	2.94 ± 0.02
TA (g/L)	10.7 ± 0.1
Alcohol (% *v*/*v*)	10.4 ± 0.0
Free SO_2_ (ppm)	11 ± 1
Total SO_2_ (ppm)	83 ± 2
A_420_ (mAU)	3.4 ± 0.1
Fructose (g/L)	1.96 ± 0.01
Glucose (g/L)	0.35 ± 0.01
Malic acid (g/L)	4.93 ± 0.08
Acetic acid (g/L)	0.11 ± 0.00

Data represent mean value ± standard deviation of base wine analyzed in triplicate.

**Table 4:** Model reaction systems prepared in Chardonnay base wine.

System ^†^	Sugar	Amino Acid	Mg^2+^ (mg/L)		Ca^2+^ (mg/L)	
CT	–	–	-	-	-	-
CT ML			10	-	-	-
CT MH			-	50	-	-
CT CL			-	-	10	-
CT CH			-	-	-	50
F	Fru 0.02 M (3603.20 mg/L)	–	-	-	-	-
F ML			10	-	-	-
F MH			-	50	-	-
F CL			-	-	10	-
F CH			-	-	-	50
FG		Gly 0.01 M (758.28 mg/L)	-	-	-	-
FG ML			10	-	-	-
FG MH			-	50	-	-
FG CL			-	-	10	-
FG CH			-	-	-	50
FL		Lys 0.01 M (1491.73 mg/L)	-	-	-	-
FL ML			10	-	-	-
FL MH			-	50	-	-
FL CL			-	-	10	-
FL CH			-	-	-	50
FC		Cys 0.01 M (1249.07 mg/L)	-	-	-	-
FC ML			10	-	-	-
FC MH			-	50	-	-
FC CL			-	-	10	-
FC CH			-	-	-	50

^†^ All conditions prepared in duplicate. Model system treatments include control (CT), fructose (F); fructose-glycine (FG), fructose-lysine (FL), and fructose-cysteine (FC) with metal additions of low (10 mg/L) Mg or Ca (ML or CL, respectively) or high (50 mg/L) Mg or Ca (MH or CH, respectively).

## Data Availability

Data available upon request.

## Supplementary Materials

The following supporting information can be downloaded at https://www.mdpi.com/article/10.3390/molecules30030535/s1: Figure S1: Sample electropherogram of cations analyzed by capillary electrophoresis. Wine sample diluted 1:10 *v*/*v*. Figure S2: Standard calibration curves for magnesium (a) and calcium (b) determination by capillary electrophoresis. Figure S3: Standard calibration curves for glycine (a), lysine (b), and cystine (c) by capillary electrophoresis. Table S1: Summary of conditions for accelerated white and model wine aging in the literature. Table S2: Three-way ANOVA results for changes in wine chemical composition (pH, TA, A_420_) during accelerated aging (at 50 °C) of modified sparkling base wine model systems for 4 weeks. Table S3: Wine chemical composition (pH, TA, A_420_) during accelerated aging (at 50 °C) of modified sparkling base wine model systems for 4 weeks, evaluated by a three-way ANOVA with Tukey’s post hoc test (*p* < 0.05). Table S4: Three-way ANOVA results for sugar composition during accelerated aging (at 50 °C) of modified sparkling base wine model systems for 4 weeks. Table S5: Glucose and fructose (g/L) composition during accelerated aging (at 50 °C) of modified sparkling base wine model systems for 4 weeks, evaluated by a three-way ANOVA with Tukey’s post hoc test. Table S6: Results of two-way ANOVA for free glycine, lysine, and cysteine assessed in their respective modified sparkling base wine model systems during accelerated aging (at 50 °C) for 4 weeks. Table S7: Aging duration*metal treatment interaction results from a two-way ANOVA with Tukey’s post hoc test for free cysteine levels (mg/L) in modified sparkling base wines treated with fructose-cysteine. Table S8: Three-way ANOVA results for calcium and magnesium composition during accelerated aging (at 50 °C) of modified sparkling base wine model systems for 4 weeks. Table S9: Calcium and magnesium (mg/L) composition during accelerated aging (at 50 °C) of modified sparkling base wine model systems for 4 weeks, evaluated by a three-way ANOVA with Tukey’s post hoc test. Table S10: Maillard reaction-associated product concentrations (mg/L) with differences according to sugar–amino acid treatments after 4 weeks of accelerated aging (at 50 °C) in modified sparkling base wine model systems. Results evaluated by a one-way ANOVA with Tukey’s post hoc test. Table S11: Maillard reaction-associated product concentrations (mg/L) with differences according to metal additions after 4 weeks of accelerated aging (at 50 °C) in modified sparkling base wine model systems. Results evaluated by a one-way ANOVA with Tukey’s post hoc test. Table S12: Mean concentrations of major trace elements in Chardonnay base wine prior to treatment additions.
