# Supplementary material for: Effect of Amino Acid, Sugar, Ca2+, and Mg2+ on Maillard Reaction-Associated Products in Modified Sparkling Base Wines During Accelerated Aging
Source: Molecules. 2025 Jan 24;30(3):535. doi: 10.3390/molecules30030535 (PMC11821130; doi:10.3390/molecules30030535)
Supplement: Supplementary file 1 [file molecules-30-00535-s001.zip › molecules-3382030-supplementary.pdf]

Supplementary Materials for:

Effect of amino acid, sugar, Ca^{2+} and Mg^{2+} on Maillard reaction-associated products in modified sparkling base wines during accelerated aging.

Hannah M. Charnock, Gary J. Pickering, and Belinda S. Kemp

Table S1. Summary of conditions for accelerated white and model wine aging in literature.

Variety	Temp (°C)	Sampling intervals	Reference
Riesling	45	0, 1, 3, 8, 16, 29, 40 days	Pickering et al., 1999
Riesling	25	6, 12, 24 months	Tarasov et al. 2021
Gewürztraminer	50	0, 2.5, 5 weeks	Carlin et al. 2022
Riesling (aroma precursor extraction; model grape must)	50	0, 1, 2, 5 weeks	Oliveira and Ferreira 2019
Chardonnay	50	7 days	Cejudo-Bastante et al. 2013
Grenache blanc and Macabeu (fortified white wine)	37	0, 3, 6, 9, 12 months	Cutzach et al. 1999
Pedro Ximenes (fortified white wine)	65	0, 10, 20, 30 days	López de Lerma et al. 2010
Model white wine	45	30 days	Clark and Scollary 2002, 2003

Table S2. Three-way ANOVA results for changes in wine chemical composition (pH, TA, A_{420}) during accelerated aging (at 50 °C) of modified sparkling base wine model systems for 4 weeks.

	pH	TA	A_{420} (mAU)
***p*-value**			
Time	***	***	***
Metal	***	n.s.	n.s.
Txmt	***	***	***
Ti x M	***	n.s.	**
Ti x Tx	***	***	***
M x Tx	***	n.s.	n.s.
Ti x M x Tx	***	n.s.	***
**F-statistic**			
Time	155.09	64.20	2302.37
Metal	191.18	1.53	0.35
Txmt	6863.26	254.95	1071.04
Ti x M	4.53	0.45	2.63
Ti x Tx	16.91	46.08	239.36
M x Tx	4.64	0.60	1.57
Ti x M x Tx	3.49	0.81	2.14
**Eta-squared (η^2)**			
Time	0.011	0.072	0.413
Metal	0.026	0.003	0.000
Txmt	0.943	0.571	0.384
Ti x M	0.001	0.002	0.002
Ti x Tx	0.005	0.206	0.172
M x Tx	0.003	0.005	0.002
Ti x M x Tx	0.004	0.015	0.006

Significance: n.s. = $p > .05$; * = $p < .05$; ** = $p < .01$; *** = $p < .001$. Ti = Time; M = Metal; Tx/Txmt = Treatment.

Table S3. Wine chemical composition (pH, TA, A_{420}) during accelerated aging (at 50 °C) of modified sparkling base wine model systems for 4 weeks, evaluated by a three-way ANOVA with Tukey's post-hoc test ($p < .05$).

Model System	Reaction time (weeks)								
	0	2	4	0	2	4	0	2	4
	pH			TA[a]			A_{420} (mAU)		
CT	2.94 ± 0.01	2.96 ± 0.02	2.96 ± 0.01	10.6 ± 0.1	10.7 ± 0.0	10.7 ± 0.1	34.3 ± 2.1	39.0 ± 0.0	44.5 ± 0.6
CT ML	2.95 ± 0.01	2.98 ± 0.01	2.97 ± 0.00	10.7 ± 0.1	10.7 ± 0.1	10.6 ± 0.1	31.8 ± 0.5	39.3 ± 1.0	47.0 ± 0.0
CT MH	2.95 ± 0.01	2.97 ± 0.01	2.95 ± 0.00	10.8 ± 0.1	10.7 ± 0.1	10.6 ± 0.1	32.5 ± 1.7	39.0 ± 0.8	46.5 ± 0.6
CT CL	2.95 ± 0.01	2.98 ± 0.01	2.97 ± 0.01	10.7 ± 0.1	10.7 ± 0.0	10.7 ± 0.1	33.0 ± 1.2	39.0 ± 0.0	45.8 ± 0.5
CT CH	2.91 ± 0.01	2.94 ± 0.01	2.92 ± 0.00	10.7 ± 0.1	10.7 ± 0.1	10.7 ± 0.1	32.3 ± 1.0	37.8 ± 0.5	48.0 ± 1.2
F	2.95 ± 0.01	2.96 ± 0.01	2.98 ± 0.01	10.7 ± 0.1	10.6 ± 0.1	10.7 ± 0.1	32.3 ± 0.5	40.5 ± 0.6	46.0 ± 1.2
F ML	2.97 ± 0.01	2.98 ± 0.01	2.99 ± 0.01	10.7 ± 0.1	10.6 ± 0.0	10.7 ± 0.1	33.3 ± 1.5	43.3 ± 1.0	47.0 ± 1.2
F MH	2.97 ± 0.01	2.96 ± 0.01	2.99 ± 0.01	10.7 ± 0.1	10.7 ± 0.1	10.7 ± 0.1	33.0 ± 1.2	39.8 ± 0.5	51.5 ± 5.2
F CL	2.98 ± 0.00	2.99 ± 0.01	2.99 ± 0.01	10.8 ± 0.1	10.7 ± 0.1	10.7 ± 0.1	32.0 ± 0.0	39.5 ± 1.7	48.0 ± 2.9
F CH	2.93 ± 0.01	2.94 ± 0.00	2.93 ± 0.00	10.7 ± 0.1	10.7 ± 0.0	10.7 ± 0.1	33.3 ± 0.5	39.8 ± 1.5	48.8 ± 1.5
FG	2.98 ± 0.01	3.02 ± 0.01	3.03 ± 0.01	10.7 ± 0.0	10.7 ± 0.0	10.7 ± 0.1	32.3 ± 0.5	63.0 ± 7.0	83.0 ± 3.5
FG ML	3.01 ± 0.01	3.02 ± 0.00	3.04 ± 0.01	10.7 ± 0.1	10.7 ± 0.1	10.7 ± 0.2	32.0 ± 0.0	59.3 ± 2.1	88.5 ± 12.7
FG MH	3.01 ± 0.00	3.01 ± 0.01	3.02 ± 0.00	10.7 ± 0.1	10.7 ± 0.0	10.7 ± 0.1	32.8 ± 1.0	55.8 ± 1.0	85.3 ± 7.8
FG CL	2.99 ± 0.01	3.00 ± 0.01	3.04 ± 0.01	10.7 ± 0.1	10.7 ± 0.1	10.7 ± 0.1	34.5 ± 1.7	56.5 ± 0.6	87.8 ± 13.6
FG CH	2.98 ± 0.01	2.99 ± 0.02	2.98 ± 0.01	10.7 ± 0.1	10.8 ± 0.1	10.7 ± 0.2	33.0 ± 1.2	54.5 ± 0.6	95.8 ± 22.8
FL	3.18 ± 0.00	3.18 ± 0.01	3.18 ± 0.01	10.7 ± 0.1	10.1 ± 0.0	10.1 ± 0.1	43.8 ± 2.1	86.5 ± 0.6	127.5 ± 0.6
FL ML	3.20 ± 0.00	3.20 ± 0.01	3.20 ± 0.02	10.6 ± 0.1	10.1 ± 0.1	10.1 ± 0.0	43.0 ± 1.8	85.5 ± 1.7	141.8 ± 9.5
FL MH	3.19 ± 0.01	3.20 ± 0.01	3.20 ± 0.01	10.7 ± 0.1	10.1 ± 0.1	10.2 ± 0.1	41.8 ± 1.0	84.0 ± 3.5	124.3 ± 6.1
FL CL	3.19 ± 0.01	3.20 ± 0.01	3.21 ± 0.01	10.6 ± 0.1	10.1 ± 0.1	10.1 ± 0.1	42.0 ± 0.8	89.3 ± 1.0	129.3 ± 4.4
FL CH	3.15 ± 0.01	3.18 ± 0.02	3.17 ± 0.01	10.6 ± 0.2	10.1 ± 0.1	10.0 ± 0.0	41.8 ± 0.5	86.5 ± 1.3	127.5 ± 1.7
FC	2.93 ± 0.01	2.96 ± 0.01	3.00 ± 0.01	10.7 ± 0.1	10.7 ± 0.1	10.7 ± 0.1	31.5 ± 0.6	65.5 ± 5.3	74.8 ± 3.2
FC ML	2.94 ± 0.01	2.96 ± 0.01	2.99 ± 0.01	10.7 ± 0.1	10.7 ± 0.0	10.7 ± 0.1	32.0 ± 1.2	59.0 ± 0.0	70.3 ± 3.8
FC MH	2.94 ± 0.01	2.95 ± 0.01	2.97 ± 0.00	10.8 ± 0.1	10.6 ± 0.0	10.7 ± 0.1	31.8 ± 0.5	71.0 ± 9.2	71.5 ± 5.3
FC CL	2.95 ± 0.00	2.97 ± 0.01	2.98 ± 0.01	10.7 ± 0.1	10.7 ± 0.1	10.8 ± 0.1	31.8 ± 1.0	60.0 ± 1.2	81.8 ± 0.5
FC CH	2.91 ± 0.00	2.91 ± 0.01	2.95 ± 0.03	10.7 ± 0.1	10.7 ± 0.1	10.7 ± 0.1	30.8 ± 0.5	61.0 ± 1.2	80.0 ± 10.4
**Time**	A	B	C	B	A	A	A	B	C

Data represents the mean value ± standard deviation of duplicate preparations of each treatment analyzed in duplicate. Model system treatments include control (CT), fructose (F); fructose-glycine (FG), fructose-lysine (FL), and fructose-cysteine (FC), with metal additions of low Mg (10 mg/L; ML), high Mg (50 mg/L; MH), low Ca (10 mg/L; CL), and high Ca (50 mg/L, CH). Unique capital letters indicate different means for each time interval (each chemical parameter assessed separately). [a] TA expressed as g/L tartaric acid equivalents.

Table S4. Three-way ANOVA results for sugar composition during accelerated aging (at 50 °C) of modified sparkling base wine model systems for 4 weeks.

	Glucose	Fructose
***p*-value**		
Time	***	***
Metal	n.s.	n.s.
Txmt	n.s.	***
Ti x M	n.s.	n.s.
Ti x Tx	***	*
M x Tx	*	n.s.
Ti x M x Tx	*	n.s.
**F-statistic**		
Time	625.20	9.88
Metal	1.45	1.01
Txmt	1.21	130159.81
Ti x M	1.68	0.23
Ti x Tx	4.67	2.50
M x Tx	1.78	0.71
Ti x M x Tx	1.69	0.22
**Eta-squared (η^2)**		
Time	0.772	0.000
Metal	0.004	0.000
Txmt	0.003	0.999
Ti x M	0.008	0.000
Ti x Tx	0.023	0.000
M x Tx	0.018	0.000
Ti x M x Tx	0.033	0.000

Significance: n.s. = p > .05; * = p < .05; ** = p < .01; *** = p < .001. Ti = Time; M = Metal; Tx/Txmt = Treatment.

Table S5. Glucose and fructose (g/L) composition during accelerated aging (at 50 °C) of modified sparkling base wine model systems for 4-weeks, evaluated by a three-way ANOVA with Tukey's post-hoc test.

Model System	Reaction time (weeks)					
	0	2	4	0	2	4
	Glucose			Fructose		
CT	0.35 ± 0.01	0.33 ± 0.02	0.30 ± 0.03	1.93 ± 0.01	1.93 ± 0.02	1.93 ± 0.04
CT ML	0.34 ± 0.02	0.32 ± 0.01	0.26 ± 0.01	1.96 ± 0.03	1.95 ± 0.03	1.95 ± 0.02
CT MH	0.35 ± 0.02	0.34 ± 0.02	0.24 ± 0.02	1.92 ± 0.02	1.92 ± 0.02	1.92 ± 0.02
CT CL	0.35 ± 0.01	0.33 ± 0.01	0.26 ± 0.02	1.95 ± 0.01	1.95 ± 0.02	1.95 ± 0.03
CT CH	0.35 ± 0.02	0.34 ± 0.01	0.24 ± 0.01	1.93 ± 0.02	1.95 ± 0.02	1.96 ± 0.04
F	0.35 ± 0.00	0.33 ± 0.01	0.25 ± 0.01	5.63 ± 0.07	5.62 ± 0.02	5.63 ± 0.03
F ML	0.36 ± 0.02	0.33 ± 0.02	0.25 ± 0.01	5.61 ± 0.03	5.61 ± 0.02	5.62 ± 0.03
F MH	0.35 ± 0.01	0.34 ± 0.02	0.24 ± 0.01	5.61 ± 0.01	5.62 ± 0.02	5.61 ± 0.01
F CL	0.35 ± 0.01	0.34 ± 0.01	0.25 ± 0.02	5.62 ± 0.03	5.62 ± 0.02	5.62 ± 0.02
F CH	0.36 ± 0.02	0.33 ± 0.03	0.25 ± 0.03	5.63 ± 0.04	5.63 ± 0.03	5.63 ± 0.06
FG	0.35 ± 0.02	0.33 ± 0.00	0.26 ± 0.01	5.64 ± 0.06	5.62 ± 0.01	5.61 ± 0.02
FG ML	0.35 ± 0.01	0.32 ± 0.01	0.27 ± 0.00	5.62 ± 0.06	5.58 ± 0.02	5.59 ± 0.02
FG MH	0.35 ± 0.01	0.34 ± 0.01	0.29 ± 0.01	5.62 ± 0.02	5.60 ± 0.02	5.61 ± 0.01
FG CL	0.34 ± 0.01	0.32 ± 0.01	0.24 ± 0.00	5.64 ± 0.01	5.58 ± 0.07	5.58 ± 0.02
FG CH	0.35 ± 0.01	0.32 ± 0.02	0.27 ± 0.01	5.63 ± 0.02	5.58 ± 0.03	5.59 ± 0.01
FL	0.37 ± 0.05	0.33 ± 0.02	0.27 ± 0.02	5.61 ± 0.06	5.58 ± 0.05	5.58 ± 0.02
FL ML	0.35 ± 0.01	0.33 ± 0.02	0.26 ± 0.02	5.60 ± 0.03	5.58 ± 0.07	5.58 ± 0.04
FL MH	0.36 ± 0.01	0.33 ± 0.01	0.29 ± 0.02	5.60 ± 0.02	5.58 ± 0.01	5.58 ± 0.06
FL CL	0.37 ± 0.02	0.32 ± 0.03	0.24 ± 0.03	5.61 ± 0.03	5.60 ± 0.01	5.59 ± 0.04
FL CH	0.37 ± 0.02	0.32 ± 0.01	0.24 ± 0.00	5.61 ± 0.05	5.58 ± 0.03	5.58 ± 0.05
FC	0.35 ± 0.02	0.30 ± 0.01	0.26 ± 0.04	5.61 ± 0.04	5.57 ± 0.03	5.58 ± 0.07
FC ML	0.36 ± 0.02	0.32 ± 0.01	0.28 ± 0.04	5.61 ± 0.01	5.57 ± 0.06	5.56 ± 0.04
FC MH	0.36 ± 0.02	0.31 ± 0.01	0.29 ± 0.05	5.59 ± 0.02	5.58 ± 0.04	5.55 ± 0.10
FC CL	0.36 ± 0.01	0.34 ± 0.03	0.27 ± 0.02	5.61 ± 0.01	5.60 ± 0.03	5.56 ± 0.01
FC CH	0.35 ± 0.01	0.33 ± 0.02	0.29 ± 0.02	5.63 ± 0.03	5.58 ± 0.02	5.55 ± 0.02
**Time**	C	B	A	B	A	A

Data represents the mean value ± standard deviation of duplicate preparations of each treatment analyzed in duplicate. Model system treatments include control (CT), fructose (F); fructose-glycine (FG), fructose-lysine (FL), and fructose-cysteine (FC), with metal additions of low Mg (10 mg/L; ML), high Mg (50 mg/L; MH), low Ca (10 mg/L; CL), and high Ca (50 mg/L, CH). Unique capital letters indicate different means for each time interval (each chemical parameter assessed separately).

Table S6. Results of two-way ANOVA for free glycine, lysine, and cysteine assessed in their respective modified sparkling base wine model systems during accelerated aging (at 50 °C) for 4 weeks.

	Glycine	Lysine	Cysteine
***p*-value**			
Time	***	***	***
Metal	**	***	***
Ti x M	n.s.	n.s.	***
**F-statistic**			
Time	429.82	12.39	989.44
Metal	3.95	7.36	17.25
Ti x M	1.34	0.91	18.13
**Eta-squared (η^2)**			
Time	0.923	0.223	0.884
Metal	0.017	0.276	0.031
Ti x M	0.012	0.068	0.065

Significance: n.s. = p > .05; * = p < .05; ** = p < .01; *** = p < .001. Ti = Time; M = Metal; Tx/Txmt = Treatment.

Table S7. Aging duration*metal treatment interaction results from a two-way ANOVA with Tukey's post-hoc test for free cysteine levels (mg/L) in modified sparkling base wines treated with fructose-cysteine.

Model System	Reaction time (weeks)	Cysteine
FC		1278.09 e
FC ML		1358.58 e
FC MH	0	1346.78 e
FC CL		1269.24 e
FC CH		1290.10 e
FC		1239.01 e
FC ML		1203.38 e
FC MH	2	1238.99 e
FC CL		601.18 c
FC CH		892.18 d
FC		113.03 a
FC ML		212.23 ab
FC MH	4	132.31 a
FC CL		161.83 ab
FC CH		354.30 b

Data represents the mean value of duplicate preparations of each treatment analyzed in duplicate. Model system treatments include fructose-cysteine (FC) with metal additions of low (10 mg/L) Mg or Ca (ML or CL, respectively), and high (50 mg/L) Mg or Ca (MH or CH, respectively). Different letters indicate that means are different, Tukey's post-hoc test.

Table S8. Three-way ANOVA results for calcium and magnesium composition during accelerated aging (at 50 °C) of modified sparkling base wine model systems for 4 weeks.

	Ca	Mg
***p*-value**		
Time	***	***
Metal	***	***
Txmt	n.s.	n.s.
Ti x M	n.s.	n.s.
Ti x Tx	n.s.	n.s.
M x Tx	n.s.	n.s.
Ti x M x Tx	***	n.s.
**F-statistic**		
Time	9.31	11.93
Metal	1679.69	812.57
Txmt	1.60	2.31
Ti x M	0.72	1.78
Ti x Tx	1.41	1.47
M x Tx	0.98	1.04
Ti x M x Tx	2.96	1.11
**Eta-squared (η^2)**		
Time	0.003	0.007
Metal	0.967	0.946
Txmt	0.001	0.003
Ti x M	0.001	0.004
Ti x Tx	0.002	0.003
M x Tx	0.002	0.005
Ti x M x Tx	0.014	0.010

Significance: n.s. = p > .05; * = p < .05; ** = p < .01; *** = p < .001. Ti = Time; M = Metal; Tx/Txmt = Treatment.

Table S9. Calcium and magnesium (mg/L) composition during accelerated aging (at 50 °C) of modified sparkling base wine model systems for 4 weeks, evaluated by a three-way ANOVA with Tukey's post-hoc test.

Model System	Reaction time (weeks)					
	0	2	4	0	2	4
	Ca			Mg		
CT	63.1 ± 0.5	60.5 ± 0.7	62.3 ± 0.3	60.6 ± 2.0	60.9 ± 2.7	60.7 ± 0.2
CT ML	62.8 ± 5.0	63.1 ± 2.7	63.4 ± 0.1	80.0 ± 4.0	71.1 ± 4.5	70.2 ± 0.6
CT MH	60.9 ± 2.2	63.5 ± 2.1	63.8 ± 0.2	106.7 ± 6.7	103.9 ± 5.0	102.6 ± 1.5
CT CL	68.0 ± 3.5	72.7 ± 2.5	70.5 ± 3.6	61.2 ± 4.5	63.3 ± 2.7	63.2 ± 0.7
CT CH	127.1 ± 2.2	124.5 ± 6.4	111.4 ± 0.1	61.9 ± 5.2	63.4 ± 3.7	57.8 ± 1.6
F	64.4 ± 1.6	62.4 ± 3.1	60.6 ± 0.5	64.4 ± 3.1	64.0 ± 3.2	59.1 ± 0.6
F ML	67.1 ± 4.3	62.7 ± 3.3	62.2 ± 1.2	74.3 ± 4.9	77.6 ± 1.8	67.1 ± 0.3
F MH	61.1 ± 3.8	64.7 ± 1.4	61.7 ± 1.5	99.3 ± 7.8	103.7 ± 7.4	97.8 ± 1.4
F CL	67.4 ± 1.6	71.5 ± 3.1	69.8 ± 2.1	61.3 ± 1.3	62.9 ± 3.3	61.1 ± 0.8
F CH	119.4 ± 6.8	125.2 ± 7.1	113.2 ± 7.5	61.8 ± 0.2	63.6 ± 2.6	61.2 ± 2.1
FG	62.7 ± 1.8	66.5 ± 2.7	59.9 ± 1.5	60.3 ± 0.1	64.8 ± 1.9	59.5 ± 3.0
FG ML	64.8 ± 4.4	62.9 ± 4.6	60.9 ± 2.4	70.2 ± 2.1	73.4 ± 0.0	66.7 ± 1.2
FG MH	66.1 ± 4.1	60.9 ± 2.0	60.5 ± 1.2	104.1 ± 2.6	111.8 ± 9.9	97.8 ± 2.0
FG CL	66.6 ± 4.9	68.4 ± 0.7	69.4 ± 0.6	59.9 ± 2.0	60.4 ± 1.5	61.0 ± 0.8
FG CH	106.9 ± 8.1	118.7 ± 3.6	118.1 ± 3.6	64.9 ± 3.4	61.7 ± 1.6	61.8 ± 1.1
FL	65.7 ± 3.4	64.3 ± 2.7	63.5 ± 3.0	63.0 ± 0.5	62.2 ± 1.5	62.6 ± 1.4
FL ML	64.9 ± 0.9	64.8 ± 1.7	60.0 ± 0.3	74.5 ± 3.4	70.9 ± 1.9	67.0 ± 1.3
FL MH	62.6 ± 2.7	65.7 ± 2.7	59.7 ± 1.2	112.5 ± 10.0	103.9 ± 3.5	96.9 ± 2.1
FL CL	73.9 ± 3.2	72.5 ± 2.7	69.1 ± 0.9	62.7 ± 2.4	61.2 ± 4.5	61.1 ± 1.2
FL CH	125.8 ± 5.4	108.6 ± 0.2	118.2 ± 0.2	59.5 ± 2.5	62.0 ± 4.4	61.0 ± 0.5
FC	61.4 ± 3.1	64.6 ± 0.8	63.3 ± 0.9	58.9 ± 1.0	62.0 ± 1.1	61.4 ± 0.9
FC ML	63.1 ± 3.0	66.6 ± 4.7	60.8 ± 3.0	67.9 ± 0.6	70.7 ± 2.2	66.3 ± 5.3
FC MH	64.1 ± 5.0	62.7 ± 3.9	58.6 ± 1.1	101.1 ± 4.1	99.7 ± 6.2	101.4 ± 7.3
FC CL	70.3 ± 0.7	67.8 ± 0.9	66.2 ± 1.5	60.2 ± 3.7	59.0 ± 0.5	58.9 ± 1.4
FC CH	120.2 ± 4.6	117.4 ± 3.3	114.6 ± 1.5	62.8 ± 3.3	60.9 ± 1.5	59.3 ± 1.2
**Time**	B	B	A	B	B	A

Data represents the mean value ± standard deviation of duplicate preparations of each treatment analyzed in duplicate. Model system treatments include control (CT), fructose (F); fructose-glycine (FG), fructose-lysine (FL), and fructose-cysteine (FC), with metal additions of low Mg (10 mg/L; ML), high Mg (50 mg/L; MH), low Ca (10 mg/L; CL), and high Ca (50 mg/L, CH). Unique capital letters indicate different means for each time interval (each chemical parameter assessed separately).

Table S10. Maillard reaction-associated product concentrations (mg/L) with differences according to sugar-amino acid treatments after 4 weeks of accelerated aging (50 °C) in modified sparkling base wine model systems. Results evaluated by a one-way ANOVA with Tukey's post-hoc test.

Model System	Benzaldehyde	2-Acetylfuran	Furfural	5-Methyl-furfural	Homo-furaneol	Furfuryl ethyl ether	Ethyl-2-furoate	2,3-Dihydro-benzofuran	Thiazole	2,3,5-Trimethyl-pyrazine
CT	11.63 a	4.44 a	100.51 b	7.07 a	3.572 b	2.08 a	21.73 a	7.07 a	2.77 a	13.22 b
F	12.24 a	4.65 a	100.05 b	7.07 a	3.505 b	2.08 a	20.84 a	7.07 a	2.64 a	12.66 b
FG	13.28 b	17.39 c	202.73 c	26.12 c	10.216 c	2.78 b	34.77 c	26.12 c	3.23 a	16.59 c
FL	13.55 b	16.39 c	248.35 d	30.56 d	12.736 d	2.77 b	32.20 b	30.56 d	3.20 a	12.62 b
FC	14.53 c	11.54 b	4.02 a	8.56 b	1.510 a	2.81 b	42.30 d	8.56 b	19.34 b	5.50 a

Data represents the mean value of each treatment. Model system sugar-amino acid treatments include control (CT), fructose (F); fructose-glycine (FG), fructose-lysine (FL), and fructose-cysteine (FC).

Table S11. Maillard reaction-associated product concentrations (mg/L) with differences according to metal additions after 4 weeks of accelerated aging (50 °C) in modified sparkling base wine model systems. Results evaluated by a one-way ANOVA with Tukey's post-hoc test.

Model System	Benzaldehyde	Homofuraneol	Furfuryl ethyl ether	2,3,5-Trimethylpyrazine
NA	12.90 a	6.30 ab	2.43 a	11.67 ab
ML	13.02 a	6.34 ab	2.41 a	11.40 a
MH	12.65 a	6.43 b	2.50 ab	12.47 bc
CL	12.84 a	6.31 ab	2.55 ab	12.38 bc
CH	13.83 b	6.17 a	2.63 b	12.66 c

Data represents the mean value of each treatment. Model system metal treatments include no metal addition (NA) and additions of low Mg (10 mg/L; ML), high Mg (50 mg/L; MH), low Ca (10 mg/L; CL), and high Ca (50 mg/L, CH). Unique letters within a column indicate different means for each model system.

Table S12. Mean concentrations of major trace elements in Chardonnay base wine prior to treatment additions.

Element	Concentration
**mg/L**	
Calcium	62 ± 4
Magnesium	60 ± 2
Manganese	0.65 ± 0.02
Potassium	403 ± 12
Sodium	9.2 ± 0.2
Strontium	0.20 ± 0.01
Zinc	0.66 ± 0.02
**mg/L**	
Aluminium	193 ± 12
Antimony	<2.0
Arsenic	12 ± 1
Barium	28 ± 1
Beryllium	<1.0
Cadmium	0.39 ± 0.02
Chromium	<10
Cobalt	<5.0
Copper	67 ± 4
Iron	<500
Lead	<5.0
Molybdenum	<10
Nickel	11 ± 1
Selenium	<5.0
Silver	<1.0
Thallium	<2.0
Tin	<5.0
Titanium	<100
Uranium	<0.5
Vanadium	<10

Data represents mean value ± standard deviation of base wine analyzed in triplicate. Values below the limit of detection for each metal are indicated as < LOD.

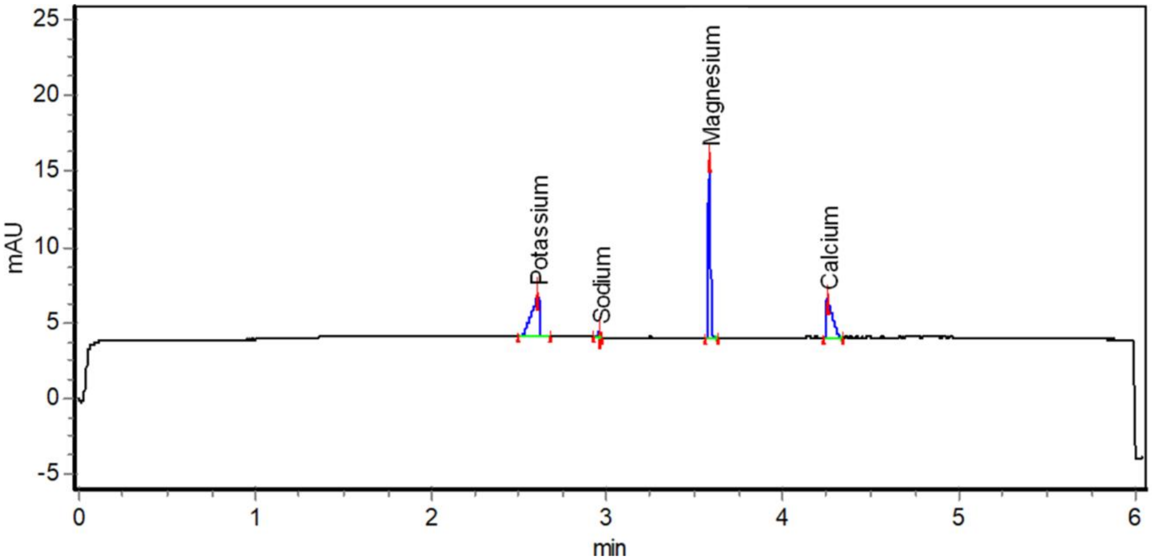

Figure S1. Sample electropherogram of cations analyzed by capillary electrophoresis. Wine sample diluted 1:10 *v*/*v*.

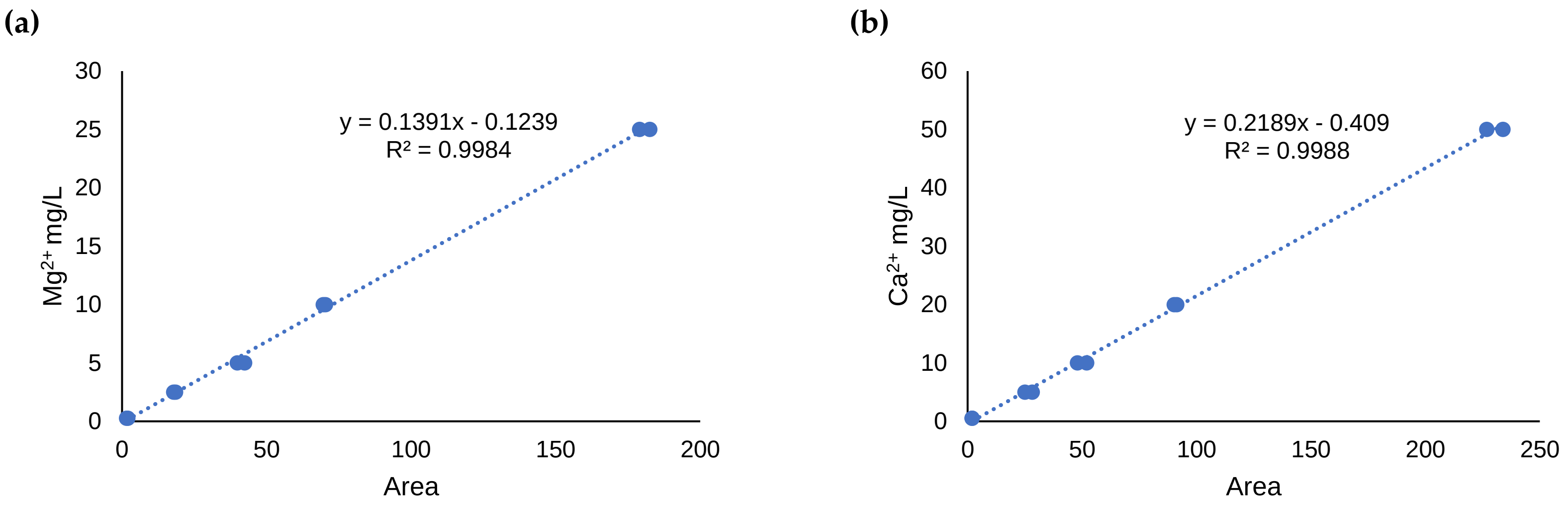

Figure S2. Standard calibration curves for magnesium (a) and calcium (b) determination by capillary electrophoresis.

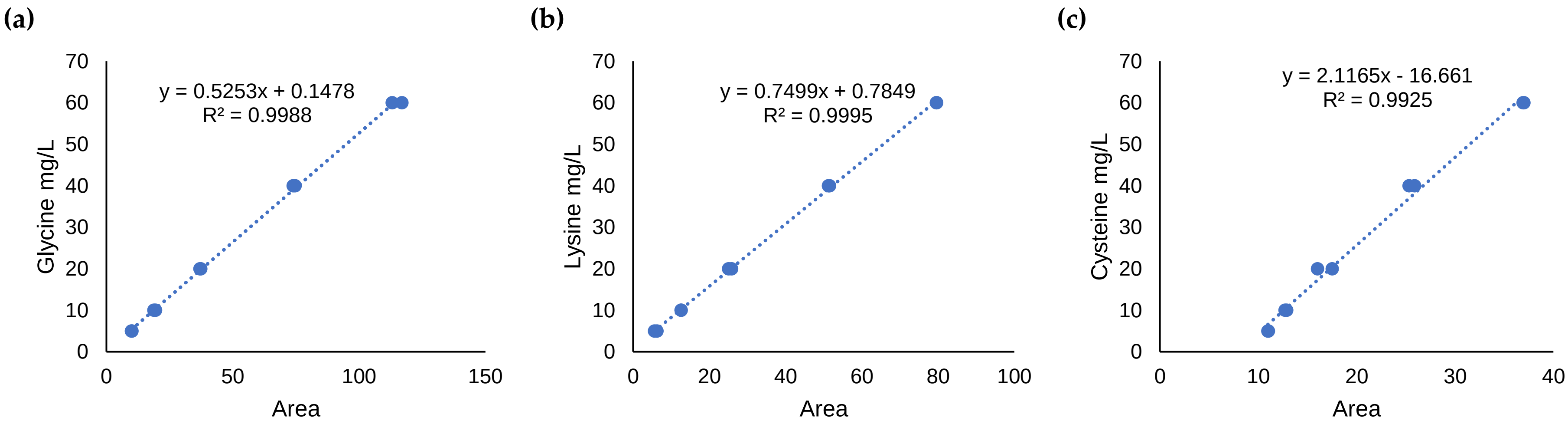

Figure S3. Standard calibration curves for glycine (a), lysine (b), and cystine (c) by capillary electrophoresis.

References: Supplementary Materials

1. Tarasov, A.; Garzelli, F.; Schuessler, C.; Fritsch, S.; Loisel, C.; Pons, A.; Patz, C.D.; Rauhut, D.; Jung, R. Wine storage at cellar vs. room conditions: Changes in the aroma composition of Riesling wine. *Molecules* **2021**, *26*, doi:10.3390/molecules26206256.
2. Carlin, S.; Lotti, C.; Correggi, L.; Mattivi, F.; Arapitsas, P.; Vrhovšek, U. Measurement of the effect of accelerated aging on the aromatic compounds of Gewürztraminer and Teroldego wines, using a SPE-GC-MS/MS protocol. *Metabolites* **2022**, *12*, doi:10.3390/metabo12020180.
3. Oliveira, I.; Ferreira, V. Modulating fermentative, varietal and aging aromas of wine using non-*Saccharomyces* yeasts in a sequential inoculation approach. *Microorganisms* **2019**, *7*, doi:10.3390/microorganisms7060164.
4. Cejudo-Bastante, M.J.; Hermosín-Gutiérrez, I.; Pérez-Coello, M.S. Accelerated aging against conventional storage: Effects on the volatile composition of Chardonnay white wines. *J. Food Sci.* **2013**, *78*, doi:10.1111/1750-3841.12077.
5. Cutzach, I.; Chatonnet, P.; Dubourdieu, D. Study of the formation mechanisms of some volatile compounds during the aging of sweet fortified wines. *J. Agric. Food Chem.* **1999**, *47*, 2837–2846, doi:10.1021/jf981224s.
6. López de Lerma, N.; Peinado, J.; Moreno, J.; Peinado, R.A. Antioxidant activity, browning and volatile Maillard compounds in Pedro Ximénez sweet wines under accelerated oxidative aging. *LWT - Food Sci. Technol.* **2010**, *43*, 1557–1563, doi:10.1016/j.lwt.2010.06.004.
7. Clark, A.C.; Scollary, G.R. Copper(II)-mediated oxidation of (+)-catechin in a model white wine system. *Aust. J. Grape Wine Res.* **2002**, *8*, 186–195, doi:10.1111/j.1755-0238.2002.tb00255.x.
8. Clark, A.C.; Scollary, G.R. Influence of light exposure, ethanol and copper(II) on the formation of a precursor for xanthylium cations from tartaric acid. *Aust. J. Grape Wine Res.* **2003**, *9*, 64–71, doi:10.1111/j.1755-0238.2003.tb00233.x.
9. Pickering, G. J.; Heatherbell, D. A.; Barnes, M. F. The production of reduced-alcohol wine using glucose oxidase-treated juice. Part II. Stability and SO2-binding. *Am. J. Enol. Vitic.* **1999**, 50(3), 299-306, doi:10.5344/ajev.1999.50.3.299.
